# Deep Learning for Population Genetic Inference

**DOI:** 10.1371/journal.pcbi.1004845

**Published:** 2016-03-28

**Authors:** Sara Sheehan, Yun S. Song

**Affiliations:** 1 Department of Computer Science, Smith College, Northampton, Massachusetts, United States of America; 2 Computer Science Division, UC Berkeley, Berkeley, California, United States of America; 3 Department of Statistics, UC Berkeley, Berkeley, California, United States of America; 4 Department of Integrative Biology, UC Berkeley, Berkeley, California, United States of America; 5 Department of Mathematics, University of Pennsylvania, Philadelphia, Pennsylvania, United States of America; 6 Department of Biology, University of Pennsylvania, Philadelphia, Pennsylvania, United States of America; Rutgers University, UNITED STATES

## Abstract

Given genomic variation data from multiple individuals, computing the likelihood of complex population genetic models is often infeasible. To circumvent this problem, we introduce a novel likelihood-free inference framework by applying deep learning, a powerful modern technique in machine learning. Deep learning makes use of multilayer neural networks to learn a feature-based function from the input (e.g., hundreds of correlated summary statistics of data) to the output (e.g., population genetic parameters of interest). We demonstrate that deep learning can be effectively employed for population genetic inference and learning informative features of data. As a concrete application, we focus on the challenging problem of jointly inferring natural selection and demography (in the form of a population size change history). Our method is able to separate the global nature of demography from the local nature of selection, without sequential steps for these two factors. Studying demography and selection jointly is motivated by *Drosophila*, where pervasive selection confounds demographic analysis. We apply our method to 197 African *Drosophila melanogaster* genomes from Zambia to infer both their overall demography, and regions of their genome under selection. We find many regions of the genome that have experienced hard sweeps, and fewer under selection on standing variation (soft sweep) or balancing selection. Interestingly, we find that soft sweeps and balancing selection occur more frequently closer to the centromere of each chromosome. In addition, our demographic inference suggests that previously estimated bottlenecks for African *Drosophila melanogaster* are too extreme.

## Author Summary

Deep learning is an active area of research in machine learning which has been applied to various challenging problems in computer science over the past several years, breaking long-standing records of classification accuracy. Here, we apply deep learning to develop a novel likelihood-free inference framework to estimate population genetic parameters and learn informative features of DNA sequence data. As a concrete example, we focus on the challenging problem of jointly inferring natural selection and demographic history.

## Introduction

With the advent of large-scale whole-genome variation data, population geneticists are currently interested in considering increasingly more complex models. However, statistical inference in this setting is a challenging task, as computing the likelihood of a complex population genetic model is a difficult problem both theoretically and computationally.

In this paper, we introduce a novel likelihood-free inference framework for population genomics by applying *deep learning*, which is an active area of machine learning research. To our knowledge, deep learning has not been employed in population genomics before. A recent survey article [1] provides an accessible introduction to deep learning, and we provide a high-level description below. Our general goal in this paper is to demonstrate the potential of deep learning as a powerful framework for population genetic analysis that can allow accurate inference of previously intractable models.

As a concrete example, we consider models of non-equilibrium demography and natural selection, for which multi-locus full-likelihood computation is prohibitive, and apply deep learning to the challenging problem of jointly inferring demographic history and selection (see [2] for a recent review of this topic). One reason why this problem is difficult is that demography (for example, a bottleneck in population size) and selection can leave similar signals in the genome. Untangling the two factors directly has rarely been attempted; most methods that estimate selection try to demonstrate robustness to demographic scenarios, rather than estimating demographic parameters jointly with selection. The demographic models considered in this paper are restricted to a single population with time-varying effective population size, but the overall framework presented here can be applied to more general demographic models. We anticipate many algorithmic, modeling, and application directions that could emerge from this proof of concept.

Our focus on the joint inference of demography and selection is motivated by *Drosophila*, where previous demographic estimates [3] may have been confounded by pervasive selection. The reverse has occurred as well, with selection estimates being confounded by demography [4]. See [5] for a more thorough discussion of the role selection plays in the *Drosophila* genome. To test our method, we simulate data under a variety of realistic demographies for an African population of *Drosophila melanogaster*. For each demographic history, we simulate many regions under different selection parameters. We then apply our tailored deep learning method using hundreds of potentially informative summary statistics. From the output of this trained network, we demonstrate that parameters can be accurately inferred for test datasets, and interpret which statistics are making the biggest contributions. After training our deep network, we also apply it to analyze 197 *Drosophila melanogaster* genomes from Zambia, Africa [6] to learn about their effective population size change history and selective landscape.

### A summary of related works

Several machine learning methods have been developed for selection only, often focusing on classifying the genome into neutral versus selected regions. Examples include methods based on support vector machines (SVMs) [7–9] or boosting [10–12]. Often these methods demonstrate robustness to different demographic scenarios, but do not explicitly infer demography.

Many methods have been developed to infer ancestral population size changes, including PSMC [13], diCal [14, 15], and MSMC [16]. These methods make the underlying assumption that selection does not significantly bias the results. This is perhaps somewhat true for humans, but is not a valid assumption for many species such as *Drosophila*, for which selection seems ubiquitous throughout the genome.

Few previous works have addressed both population size changes and selection. Galtier *et al.* [17] developed a likelihood-based method for distinguishing between a bottleneck and selection. They applied their method to *Drosophila* data to conclude that a series of selective sweeps was more likely than a bottleneck, but did not explicitly infer parameters for both selection and demography. In their method, Galtier *et al.* assumed that demographic events affect the entire genome, whereas selection is a local phenomenon. In contrast, Gossmann *et al.* [18] estimated the effective population size locally along the genome, and reported that it is correlated with the density of selected sites. To make our results as easily interpretable as evolutionary events as possible, we estimate global effective population size changes.

Approximate Bayesian Computation (ABC) [19, 20] is a likelihood-free inference method based on simulating datasets and comparing their summary statistics. (A more detailed description of the framework is provided below.) This approach has been used to study various complex population genetic models (e.g., [21–23]) for which likelihood computation is prohibitive. Partly due to several influential theoretical works [24–31], the popularity of ABC has grown rapidly over the past decade. ABC’s main advantages are that it is easy to use and is able to output a posterior distribution. There are a few challenges, however: 1) ABC suffers from the “curse of dimensionality,” with decreasing accuracy and stability as the number of summary statistics grows [32]. 2) ABC typically uses a rejection algorithm, so the simulated datasets are not used optimally, and often a very large number are required. 3) It is difficult to interpret the output of ABC in terms of which statistics were the most informative for each parameter. 4) Although ABC can be used very effectively for continuous parameter inference, it would be difficult to use ABC for a complex scenario that involved joint inference of continuous parameters and categorical distributions. To our knowledge, ABC has thus not been previously applied to the problem of jointly inferring demography (using continuous effective population sizes) and selection (using discrete classification). The work of Bazin *et al.* [33] estimates demographic and selection parameters using a hierarchical ABC approach, though not in an explicit joint framework.

We see our deep learning method as a complimentary approach to, rather than a direct replacement of, other likelihood-free inference methods such as ABC or SVM. Deep learning has several advantages such as making full use of datasets, elegantly handling correlations between summary statistics, and producing interpretable features of the data. It also produces an “inverse” function from the input summary statistics to the population genetic parameters of interest. However, in contrast to ABC, it does not provide a posterior distribution.

### A brief introduction to deep learning

Deep learning has its beginnings in neural networks, which were originally inspired by the way neurons are connected in the brain [34]. Neural networks have been studied for over 60 years and a huge body of literature exists on the topic. A neural network is typically used to learn a complex function between inputs (data) and outputs (*response variables*) in the absence of a model, so it can be thought of as a generalized regression framework. While standard regression and classification methods involve fitting linear combinations of *fixed* basis functions, a neural network tries to *learn* basis functions (usually non-linear) appropriate for the data. A neural network architecture consists of multiple layers of *computational units* (nodes), with connections between the layers but not between nodes within a layer. Within a layer, each node computes a transformation (usually non-linear) of the outputs from the previous layer. S1 Fig illustrates a simple feed-forward neural network with a single hidden layer. The phrase “deep learning” refers to algorithms for learning *deep* neural network architectures with many hidden layers.

The *universal approximation theorem* [35, 36] states that any continuous function on compact subsets of $R^{n}$ can be uniformly approximated by a feed-forward neural network with a single hidden layer, provided that the number of nodes in the hidden layer is sufficiently large and the transformation (called the activation function) associated with each node satisfies some mild conditions. However, it can be challenging to learn the weights of such a network and to interpret the hidden layer. So as learning problems became more complex, it was desirable to train networks with more hidden layers. Since their introduction over 30 years ago, deep architectures have proved adept at modeling multiple levels of abstraction. However, they were notoriously difficult to train since their objective functions are non-convex and highly non-linear, and the level of non-linearity increases with the number of layers in the network.

A major breakthrough was made in 2006 when Hinton and Salakhutdinov [37] showed that a deep feed-forward neural network can be trained effectively by first performing unsupervised “pretraining” one layer at a time, followed by supervised fine-tuning using a gradient-descent algorithm called *back-propagation* [38]. (Simply put, pretraining provides a good initialization point for non-convex optimization.) They applied their learning algorithm to dimensionality reduction of images and achieved substantially better results than PCA-based methods.

Following the work of Hinton and Salakhutdinov, deep learning has been applied to various challenging problems in computer science over the last several years, making groundbreaking progress. Deep learning broke long-standing records for accuracy that had been set by approaches based on hand-coded rules. Well-known examples include automatic speech recognition (transforming spoken words into typed text) [39, 40] and computer vision (automatically classifying images into different categories and tagging objects/individuals in photos) [41].

Many variations have been developed, including *dropout*, which attempts to learn better and more robust features of the data (see [42, 43]). Deep learning has also been applied to problems in neuroscience [44] and computational biology [45–48], but has not been used for population genetics before. We demonstrate here that deep learning can accommodate more complex models than existing techniques, and can provide a complimentary approach to existing likelihood-free inference in population genetics. For example, deep learning could be used to select optimal statistics for ABC (as demonstrated in [49]), or another method.

### ABC background

Rejection-based methods have been used since the late 1990’s (see [19, 50]) to estimate population genetic parameters when the likelihood is difficult to compute. Early improvements to ABC quickly helped make it a popular method for a variety of scenarios (see [20] for a good introduction). ABC works by simulating many datasets under a prior for the parameters of interest. Then these datasets are reduced to a vector of summary statistics that are ideally informative for the parameters. The summary statistics that are closest to the summary statistics for the target dataset are retained, and the corresponding parameters used to estimate the desired posterior distributions. ABC has many advantages, including ease of use and the output of a posterior distribution. However, there are a few shortcomings, which various works have sought to address.

In its simplest form, ABC does not naturally handle correlated or weakly informative summary statistics, which can add noise to the data. To tackle this problem, many methods for dimensionality reduction or wisely selecting summary statistics have been proposed (see [26, 29, 31, 51, 52], and [53] for a dimensionality reduction comparison). However, simple reductions cannot always learn subtle relationships between the data and the parameters. Expert pruning of statistics helps some methods, but given the lack of sufficient statistics, valuable information can be eliminated. Blum and François [28] suggested performing a dimensionality reduction step on the summary statistics via a neural network similar to S1 Fig. This reduction is similar in spirit to the work presented here, although there are many algorithmic and application differences.

A second issue with ABC is the rejection step, which does not make optimal use of the datasets that are not retained. The more statistics and parameters used, the more datasets must be simulated and rejected to properly explore the space, making the interaction between these two issues problematic. To address the problem of rejecting datasets, different weighting approaches have been proposed (see [28] for a good example of how the estimation error changes as fewer datasets are rejected). The idea is to keep more datasets, but then weight each retained dataset by its distance to the target dataset. However, few approaches utilize all the datasets in this way, and the most popular implementation of ABC (*ABCtoolbox*, [54]) typically still rejects most of the simulated datasets by default.

One final issue with ABC is the black-box nature of the output. Given the distances between the simulated datasets and the target dataset, and the posterior, there is no clear way to tell which statistics were the most informative.

## Results

We first describe the simulated dataset we generated to investigate joint inference of demographic history and selection. In the Methods section, we describe how to transform each region (as well as each region of the real data) into a set of 345 summary statistics, and also detail our deep learning algorithm. In what follows, we present the results of our method in a variety of different contexts.

### Simulating data

**Fig 1:**
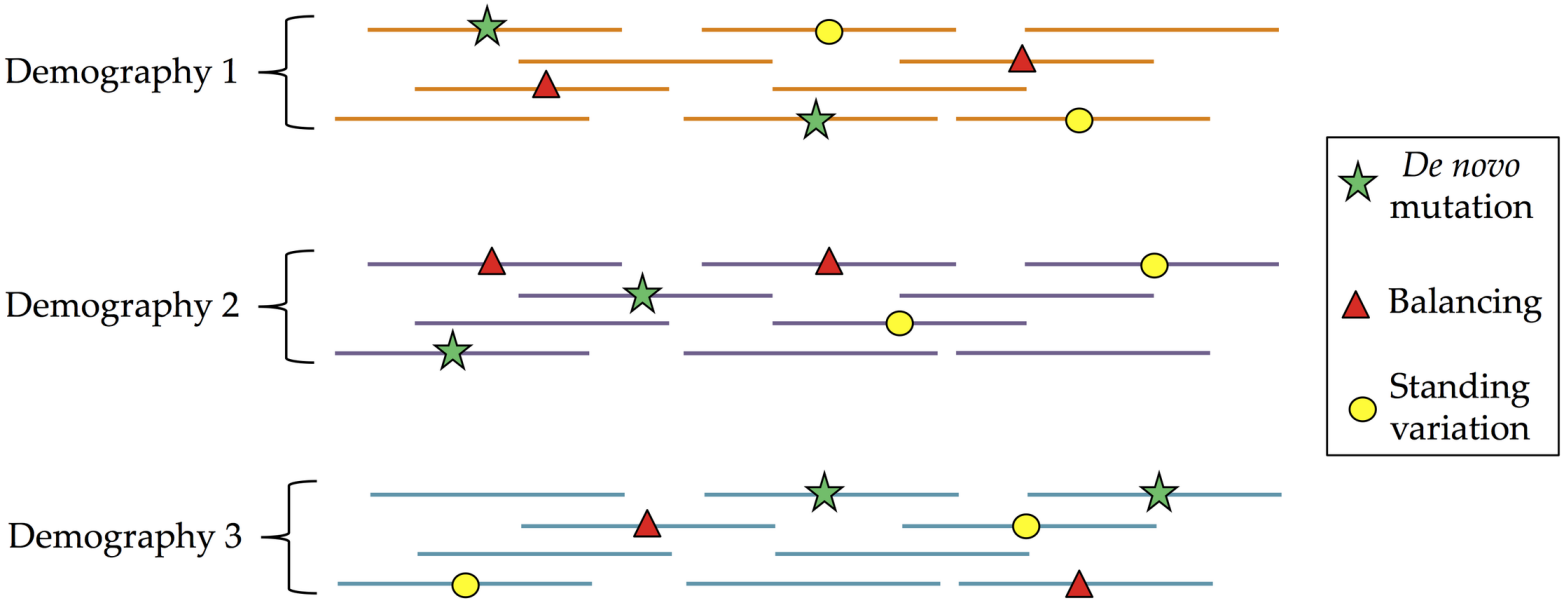
Input data for the demography and selection scenario. For each demographic history (bottleneck), we simulated many different genomic regions. Each region can either have no selection (neutral), one site with an *de novo* mutation under positive selection (hard sweep), one site under balancing selection, or one standing variant under positive selection (soft sweep).

To create a simulated dataset that is appropriate for our scenario of interest, we first define the response variables we would like to estimate. For simplicity, we consider piecewise-constant effective population size histories with three epochs: a recent effective population size *N*_1_, a bottleneck size *N*_2_, and an ancient size *N*_3_. Further, we define a genomic region as belonging to 4 different selection classes: no selection (neutral), positive directional selection (hard sweep), selection on standing variation (soft sweep), and balancing selection. See [55] for a more complete analysis of the different types of selection in *Drosophila*. To accurately reflect that demography affects the entire genome while selection affects a particular region, we simulate many genomic regions under the same demographic history, but the selection class for each region is chosen independently. See Fig 1 for a simplified illustration of the data.

To simulate data, we used the program msms [56]. To make our simulated data as close to the real data as possible, we simulated *n* = 100 haplotypes, and correspondingly downsampled the Zambia *Drosophila melanogaster* dataset to match. We repeated the following procedure 2500 times. First we selected three population sizes for the demographic model, then simulated 160 regions with these sizes, 40 for each selection scenario. Each region was 100 kb, with the selected site (if present) occurring randomly in the middle 20 kb of the region. We used a baseline effective population size *N*_ref_ = 100,000, a per-base, per-generation mutation rate *μ* = 8.4 × 10^−9^ [57], and a per-base, per-generation recombination rate *r* equal to *μ*, as inferred by PSMC [13]. This ratio of recombination to mutation rate is somewhat lower than what has been inferred in other works (see [58, 59]), so we also simulated a testing dataset with a higher recombination rate, described below. However, it is likely that there is significant variation in this ratio across the genome [60]. We used a generation time of 10 generations per year. Based on the population size change results of PSMC, we chose the time change-points for demographic history to be *t*_1_ = 0.5 and *t*_2_ = 5 in coalescent units. Scaled effective population size parameters λ_*i*_ : = *N*_*i*_/*N*_ref_ and their prior distributions are below:

1. Recent effective population size scaling factor: λ_1_ ∼ Unif(3, 14).
2. Bottleneck effective population size scaling factor: λ_2_ ∼ Unif(0.5, 6).
3. Ancient effective population size scaling factor: λ_3_ ∼ Unif(2, 10).

For the selection classes, the different types are shown below:

- **Class 0, neutral**: no selection, neutral region.
- **Class 1, hard sweep**: positive selection on a *de novo* mutation (i.e., hard sweep). For the selection coefficient, we used *s* ∈ {0.01, 0.02, 0.05, 0.1}, with 10 regions for each value of *s*. The onset of selection was chosen to be 0.005 in coalescent units, which provided sweeps at a variety of different stages of completion at the present time. We discarded datasets where the frequency of the selected allele was 0 (early loss of the beneficial mutation due to drift), but for a large fraction of datasets, the beneficial allele had not yet fixed.
- **Class 2, soft sweep**: positive selection on standing variation (i.e., soft sweep). The selection coefficients and selection start time were chosen as in the hard sweep scenario, but now the initial frequency of the beneficial allele was chosen to be 0.001.
- **Class 3, balancing**: heterozygote advantage, balancing selection. The selection start time and selection coefficients were chosen in the same fashion as Class 1.

Using this strategy, we simulated 2500 different demographic histories, with 160 regions for each one, for a total of 400,000 datasets. To build 160 regions for each demography, we simulated 40 datasets for each of the selection classes above.

### Results for demography and selection on simulated data

Of the 400,000 total datasets, we used 75% for training and left out 25% for testing. In Table 1, we show the effective population size results for a network with 3 hidden layers respectively with 25, 25, and 10 nodes (5 layers total including input and output). The best results were found when we averaged the statistics for all of the datasets (160 “regions”) with the same demography, then ran this average through the trained network.

**Table 1:** Deep learning results for population sizes under the scenario with demography and selection, using a network with 3 hidden layers of sizes 25, 25, and 10. We evaluate the results in three ways, using the relative error of the estimates: |*N*_est_ − *N*_true_|/*N*_true_. First, for each test demography, we average the statistics for each dataset, and then run these values through the training network. Second, for each test demography, we run the datasets through the network one by one, then average the predictions. Finally, we perform the second procedure, but with only the regions we classified as neutral. We note that the most ancient size (*N*_3_) is always the least accurately estimated.

Deep learning predictions	*N*_1_ error	*N*_2_ error	*N*_3_ error
Average stat prediction	0.051	0.074	0.487
Final prediction	0.098	0.077	0.569
Neutral regions prediction	0.072	0.083	0.566

**Fig 2:**
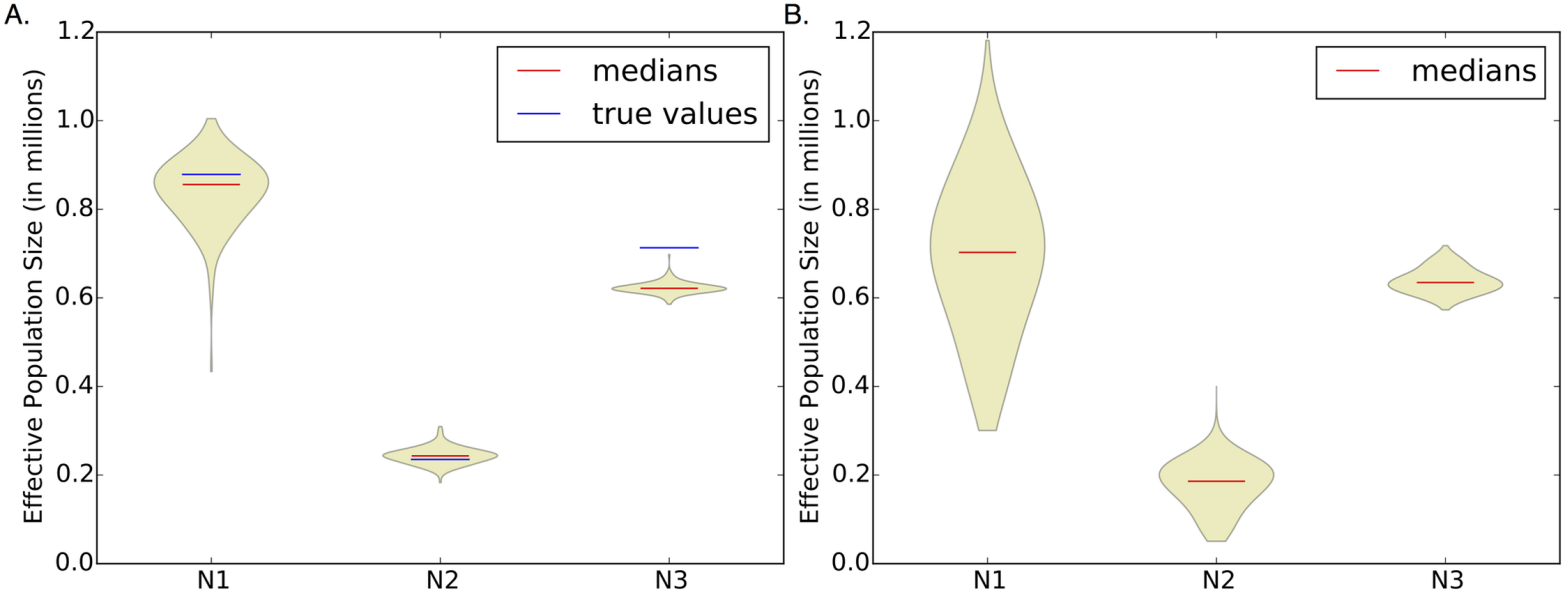
Violin plots of population size estimates for (A) an example simulated dataset and (B) the real dataset. In both cases, we have the largest uncertainty for the most recent population size. For the simulated dataset, the true population sizes are shown in blue, and we note that our point estimate of the most ancient population size is the least accurate.

Table 2 shows the results of our three different size prediction methods (see Methods) for an example demography. For each parameter, we include a range between the 2.5th and 97.5th quantiles. To further investigate uncertainty in our estimates, we also include a violin plot of the three population sizes, shown in Fig 2A. In this example (and more generally), our point estimate of the most ancient size *N*_3_ is the least accurate, but the most recent size *N*_1_ has the largest uncertainty. When considering a single dataset, there is not always a clear winner among our three prediction methods. Overall, the average statistic method is usually the most accurate, followed by the neutral regions method.

**Table 2:** Results for an example demography, with the true population sizes shown in the first row. The rows below each dotted line are the three different size predictions for this dataset. *Average stat*: average the statistics for each region within the same demography, then run these averages through the trained network. *Final*: run the statistics for each region through the trained network, then average the results. *Neutral regions*: only average the results for the regions we predict as neutral.

	*N*_1_	*N*_2_	*N*_3_
True sizes	878,675	235,199	713,001
Average stat prediction	872,541	264,817	600,650
Final prediction	840,784	243,180	622,359
Quantiles (2.5th, 97.5th)	(659231, 954631)	(209797, 279496)	(597402, 649887)
Standard deviation	74,477	37,238	54,165
Neutral regions prediction	873,289	237,686	619,943

To analyze the selection results, we calculated a confusion matrix in Table 3 to show which datasets of each class were classified correctly, or misclassified as belonging to a different class. The rows of a confusion matrix represent the true class of each dataset, and the columns represent the predicted or called class. An ideal confusion matrix would have all 1’s (100%) on the diagonal, and 0’s off the diagonal. Our most frequent error is classifying hard sweep datasets as neutral (row 2, column 1 of the confusion matrix). We hypothesized that this was either because selection occurred anciently and quickly, or because selection occurred recently and the sweep was not yet complete. To test this, we examined the results conditional on the frequency of the selected allele at present. The results shown in Table 4 suggest that in fact many of the sweeps were not complete, which is why the regions sometimes appeared neutral. Regardless, this type of false negative error is arguably preferable to falsely calling many regions to be under selection when they are in fact neutral.

**Table 3:** Confusion matrix for the selection predictions, in the demography and selection scenario. Each row represents the datasets that truly belong to each selection class. Each column represents the datasets that were actually classified as each selection class. Ideally we would like all 1’s down the diagonal, and 0’s in the off-diagonal entries. The largest number in each row is shown in boldface. We can see that neutral datasets are the easiest to classify, and sometimes regions under selection (hard sweeps in particular) look neutral as well (first column). The overall percentage of misclassified datasets was 6.2%.

	Called Class			
True Class	Neutral	Hard Sweep	Soft Sweep	Balancing
Neutral	**0.9995**	0.0002	0.0003	0.0000
Hard Sweep	0.1434	**0.8333**	0.0032	0.0201
Soft Sweep	0.0096	0.0010	**0.9891**	0.0003
Balancing	0.0301	0.0356	0.0056	**0.9287**

**Table 4:** Hard sweep results, broken down by the present-day frequency (*f*) of the selected allele (which we would not know for a real dataset). We defined “low” frequency as *f* ∈ [0, 0.3), “moderate” frequency as *f* ∈ [0.3, 0.7), and “high” frequency as *f* ∈ [0.7, 1]. We can see that if the frequency of the selected allele is low, the region is often misclassified as neutral, since the selective sweep is not yet complete. However, if the frequency is moderate or high, the dataset is usually classified correctly as a hard sweep.

		Called Class			
*f*		Neutral	Hard Sweep	Soft Sweep	Balancing
Low	(18.4% of datasets)	**0.6820**	0.3137	0.0009	0.0035
Moderate	(12.5% of datasets)	0.0988	**0.8220**	0.0000	0.0793
High	(69.1% of datasets)	0.0084	**0.9734**	0.0044	0.0138

We also wanted to test the impact of unsupervised pretraining using autoencoders (see Methods). Tables 5 and 6 compare the results for a randomly initialized network to a network initialized using autoencoders. These results demonstrate that pretraining is very effective. Due to the non-convex nature of the optimization problem, random initialization is most likely finding a poor local minima for the cost function.

**Table 5:** Relative error on the test dataset, for a deep network with 6 hidden layers. For the results in the first row, the weights of the entire network were initialized randomly, then optimized. In the second row, the weights were initialized using autoencoders for each layer. The positive impact of unsupervised pretraining is clear; random initialization causes the optimization to get stuck in a local minima.

Initialization Type	*N*_1_ error	*N*_2_ error	*N*_3_ error
Random	0.429	0.421	0.710
Autoencoder	0.061	0.166	0.577

**Table 6:** Confusion matrix for the selection predictions, compared between random initialization (top) and autoencoder initialization (bottom), for a deep network with 6 hidden layers. Again, ideally we would like all 1’s down the diagonal, and 0’s in the off-diagonal entries. The largest number in each row is shown in boldface. When the network is initialized randomly, almost every dataset is classified as neutral; the network has not really learned anything meaningful from the input data. The overall percentage of misclassification is 74.8% for random initialization, while it is only 6.1% for autoencoder initialization.

		Called Class		
True Class	Neutral	Hard Sweep	Soft Sweep	Balancing
		Random Initialization		
Neutral	**1.000**	0.000	0.000	0.000
Hard Sweep	**0.978**	0.007	0.000	0.015
Soft Sweep	**1.000**	0.000	0.000	0.000
Balancing	**1.000**	0.000	0.000	0.000
		Autoencoder Initialization		
Neutral	**1.000**	0.000	0.000	0.000
Hard Sweep	0.145	**0.831**	0.004	0.021
Soft Sweep	0.011	0.001	**0.987**	0.000
Balancing	0.030	0.028	0.001	**0.941**

Lastly, we wanted to assess the impact of training the deep network on data that was created under different conditions than the testing data. In particular, we simulated testing data where the ratio of the recombination rate to the mutation rate was equal to 4 (instead of 1 as in our previous results). The results of this experiment are shown in S1 Table. This rather large difference in the testing data negatively impact the results, but much more so for selection than for the effective population sizes. This is perhaps due to the dependence of selection inference on LD and haplotype statistics, which would be more affected by recombination rate variation.

We also simulated testing data with bottlenecks that were more extreme than the training data, to assess the impact of a misspecified range of demographic models. Specifically, the most recent population size was outside the range of the training data for all the testing datasets. The results are shown in S2 Table. In contrast to the previous testing scenario, here the population size results are somewhat negatively impacted (largely for the most recent size), but the selection results are only impacted slightly. In general, identifying model misspecification for continuous population genetic parameters can be done as follows: if the predicted value of a *normalized* continuous parameter is outside the range [0,1], then the testing/real data parameter is outside the range of the training/simulated data parameter. This simple criterion can help users identify when their simulated data needs to be modified to provide accurate results for their real data.

### Results on real *Drosophila melanogaster* data

We then ran the real *Drosophila melanogaster* data through our trained network just like any other test dataset. We considered only the chromosome arms 2L, 2R, 3L, and 3R in this study. We partitioned each chromosome arm into 20 kb windows and ran our method on five consecutive windows at a time, sliding by 20 kb after each run. Classification was performed on the middle 20 kb window. The population size predictions for some of these regions (roughly 14%) were outside the range of the simulated data, so we discarded them from all further analysis. These regions tended to be near the telomeres and centromeres, where assembly is often poor.

**Fig 3:**
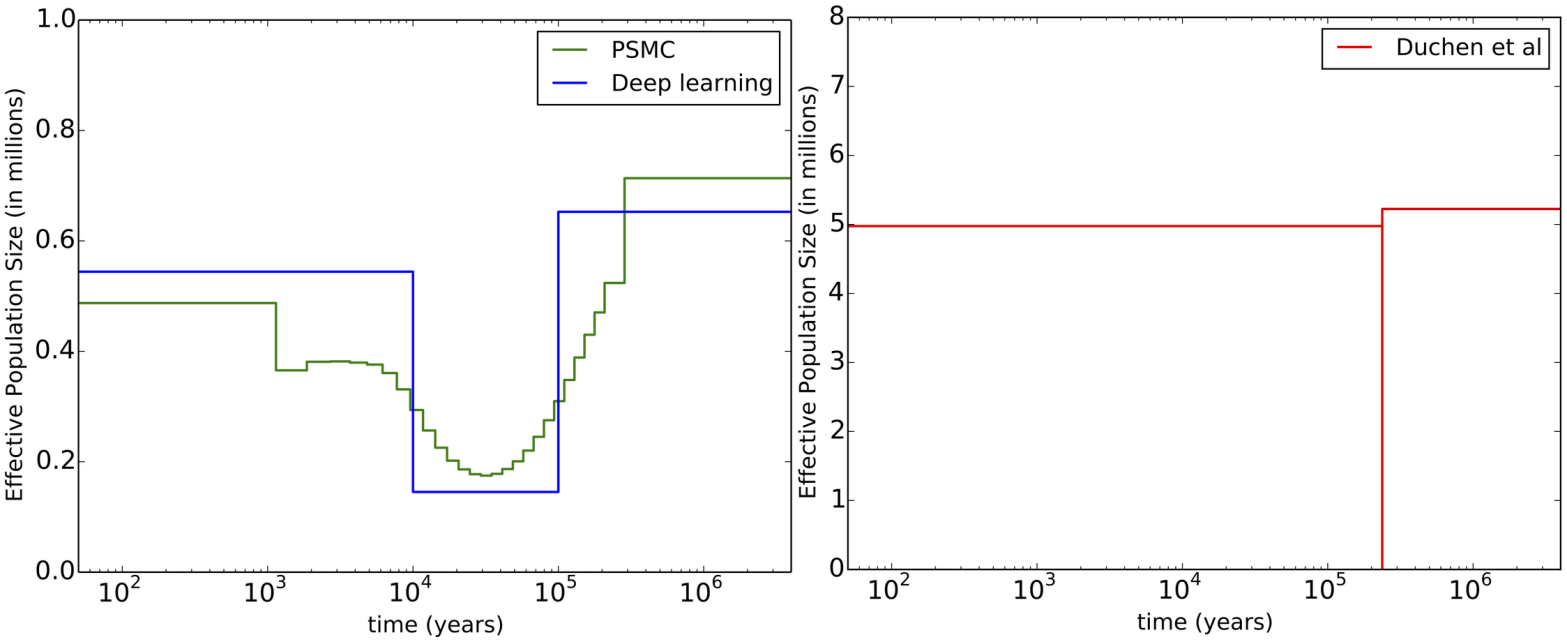
Comparison between demographic histories. On the left in green is PSMC [13], run on the entire genome for a subset of the data (*n* = 20). In blue is our history from the first line of Table 7. On the right in red is the history from Duchen *et al.* [3] (note the change in the *y*-axis scale). The difference between our estimates and these previous estimates is likely due in large part to their assumption of a very short (1000 generations) bottleneck. Given this timing constraint, only a very severe bottleneck could fit the data. A more gradual bottleneck seems more realistic, although we do not have a simple explanation for why there was a bottleneck at all.

For the demographic history, the population size results are shown in Table 7. The first row (average statistic method) is our best estimate, which we also plot and compare with other histories in Fig 3. Our history is close to the PSMC result, although more resolution would be needed for a proper comparison. We based our time change points (*t*_1_ and *t*_2_) on PSMC, so it would be surprising if these histories were notably inconsistent. The expansion after the bottleneck is roughly consistent with previous results citing the range expansion of *Drosophila melanogaster* (out of sub-Saharan Africa) as beginning around 15,000 years ago [61]. This range expansion likely led to an effective population size increase like the one we infer. Our expansion time is also consistent with bottleneck estimates in non-African *Drosophila melanogaster* populations, such as the Netherlands population investigated by Thornton and Andolfatto [58]. In Fig 2B, we include a violin plot of the population size estimates from each region. We have the largest uncertainty for the most recent population size.

**Table 7:** Population size results for African *Drosophila* from Zambia, rounded to the nearest hundred. The first row (predictions based on averaging the statistics for each region in the *Drosophila* genome) represents our best estimate of the population sizes.

Prediction	*N*_1_ (recent)	*N*_2_ (bottleneck)	*N*_3_ (ancestral)
Average stat prediction	544,200	145,300	652,700
Final prediction	694,000	178,400	638,300
Quantiles (2.5th, 97.5th)	(359300, 1004300)	(63800, 265000)	(593200, 694900)
Standard deviation	170,400	53,800	27,100
Neutral regions prediction	635,900	170,700	646,700

**Fig 4:**
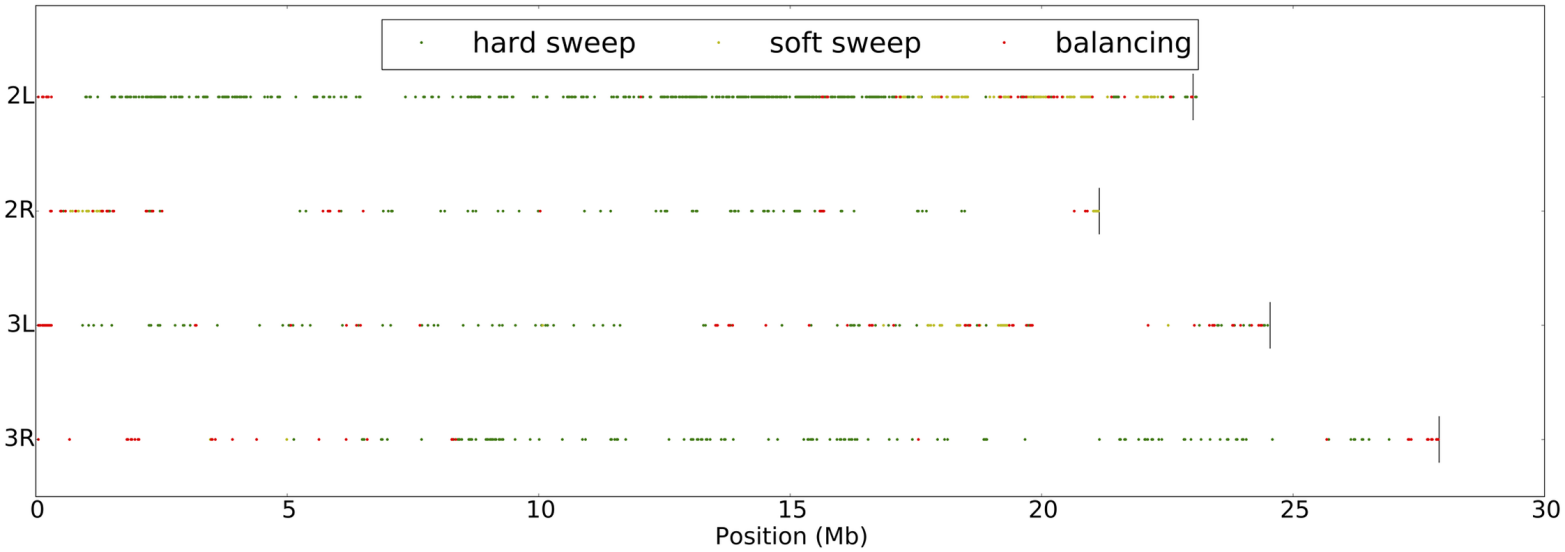
Plot of the selected regions we infer for African *Drosophila melanogaster*. Each of the 4 subplots is a chromosome arm (2L, 2R, 3L and 3R). We restricted the plot to selected sites with a probability of at least 0.95.

The number of windows classified as Neutral, Hard Sweep, Soft Sweep, and Balancing Selection are 1101, 2455, 179, 404, respectively. See S3 Table for further details. If we restrict our analysis to regions classified with probability greater than 0.9999, then we find 42 hard sweeps, 34 soft sweeps, and 17 regions under balancing selection. In S4 Table, we include a table of these high-confidence windows, along with which genes are found in each window. We also include a plot, Fig 4, of where the selected regions fall throughout the genome (restricted to regions with a probability at least 0.95). Interestingly, soft sweeps and balancing selection seem to occur more frequently closer to the centromere of each chromosome. There is also an excess of hard sweeps on chromosome arm 2L.

Upon examining the genes in the top regions classified to be under selection, we find several notable results. In the hard sweep category, a few of the top regions harbor many genes involved in chromatin assembly or disassembly. Further, there are two regions each containing exactly one gene, where the gene has a known function. These are *Fic domain-containing protein* (detection of light and visual behavior), and *charybde* (negative regulation of growth).

In the soft sweep category, we find many genes related to transcription, and several related to pheromone detection (chemosensory proteins). We also find four regions each containing exactly one gene, with that gene having a known function. These are *gooseberry* (segment polarity determination), *steppke* (positive regulation of growth), *Krüppel* (proper body formation), and *Accessory gland protein 36DE* (sperm storage, [62]). Fly embryos with a mutant *Krüppel* (German for “cripple”) gene have a “gap” phenotype with the middle body segments missing [63]. We also identify three interesting genes in regions with other genes: *seminal fluid proteins 36F* and *38D* (see [64]), and *deadlock*, which is involved in transposable element silencing, which preserves genome integrity [65].

Finally in the balancing selection category, one interesting gene we find is *nervana 3*, which is involved in the sensory perception of sound. We also find balancing selection regions containing the genes *cycle* (circadian rhythms) and *Dihydropterin deaminase* (eye pigment), although there are other genes within these putatively selected regions.

### Most informative statistics

One of the advantages of deep learning is that it outputs an optimal weight on each edge connecting two nodes of the network, starting with the input statistics and ending with the output variables. By investigating this network, we are able to determine which statistics are the “best” or most helpful for estimating our parameters of interest. Here we use two different methods, one using permutation testing and the other using a perturbation approach described in S1 Algorithm.

**Fig 5:**
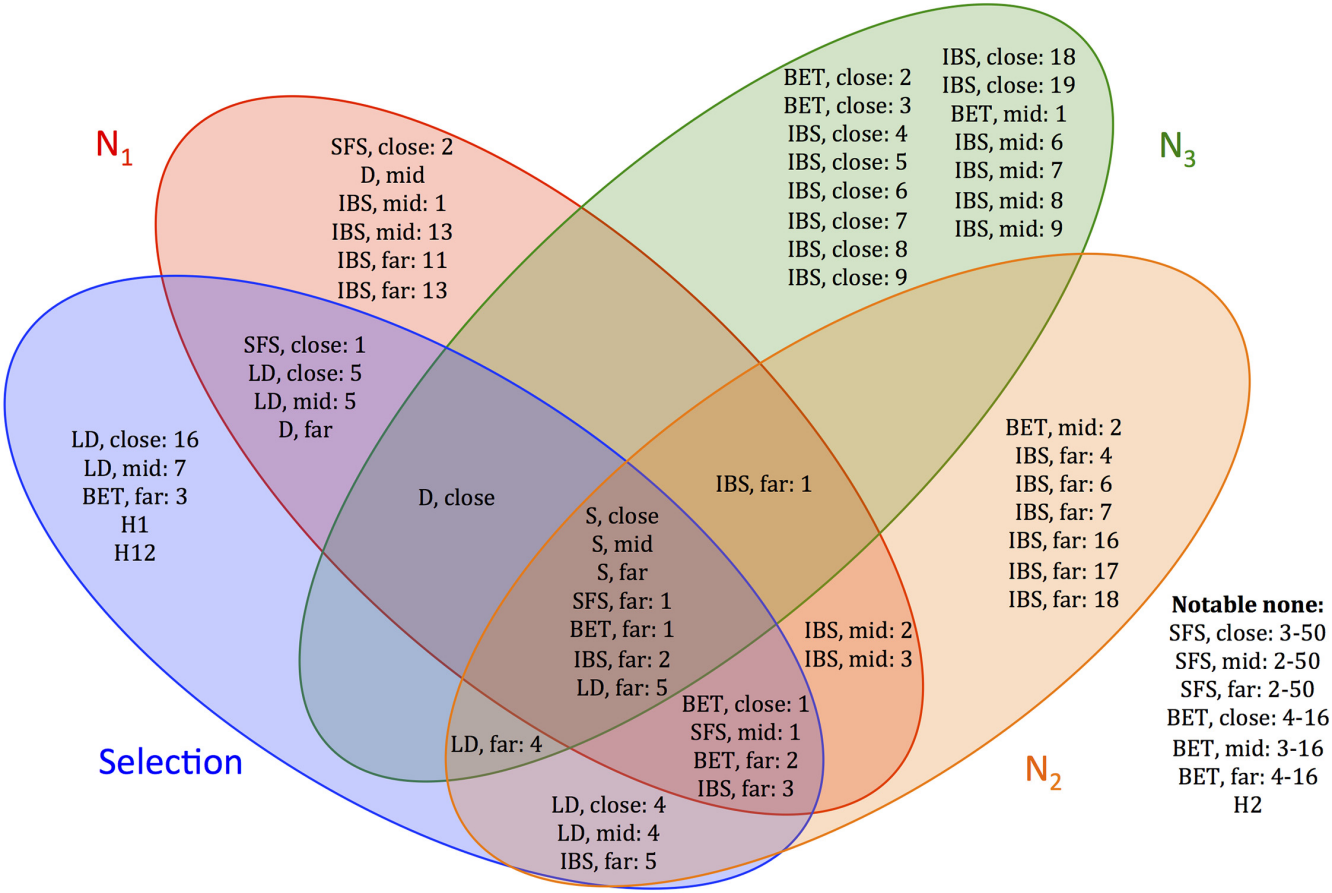
A Venn diagram of most informative statistics for each output variable (*N*_1_, *N*_2_, *N*_3_, and selection). For each variable, the top 25 statistics were chosen using permutation testing. The Venn diagram captures statistics common to each subset of output variables, with notable less informative statistics shown in the lower right. Close, mid, and far represent the genomic region where the statistic was calculated. The numbers after each colon refer to the position of the statistic within its distribution or order. For the SFS statistics, it is number of minor alleles. For each region, there are 50 SFS statistics, 16 BET statistics (distribution between segregating sites), 30 IBS statistics, and 16 LD statistics.

To perform permutation testing, we randomly permuted the values of each statistic across all the test datasets and then measured the resulting decrease in accuracy. For each output (3 population sizes and selection category), we then retained the 25 statistics that caused the largest accuracy decreases. The results are shown in the 4-way Venn diagram in Fig 5, which highlights statistics common to each subset of outputs. For each statistic name in the diagram, *close*, *mid*, and *far* represent the genomic subregion where the statistic was calculated. The numbers after each colon refer to the position of the statistic within its distribution or order; for the folded site frequency spectrum (SFS) statistics, it is the number of minor alleles. Several statistics are informative for all the parameters, including the raw number of segregating sites (“S”) in each subregion and the number of singletons (“SFS, far: 1”). There is generally more overlap between the statistics for selection and *N*_1_ or *N*_2_, than with the statistics for selection and *N*_3_, possibly because selection occurs on a more recent timescale. Tajima’s D is also most important for selection (which it was designed to detect) and *N*_1_. Identity-by-state (IBS) statistics seem to play an increasingly more important role as the population sizes become more ancient. Perhaps unsurprisingly, LD statistics are most important for selection. Interestingly, the SFS is not as informative as one might have anticipated. However, the number of singletons is very informative, especially for selection and *N*_1_, which could have been foreseen given that recent events shape the terminal branches of a genealogy, and thus the singletons. The distances between segregating sites (“BET” statistics) do not generally seem very helpful. Neither does H2, the frequency of the second most common haplotype. It is comparing H1 to H2 (via the H12 statistic) that is helpful.

The results (S2 Fig) from the perturbation method share some similarities and have some differences from the Venn diagram in Fig 5. An interesting set of four statistics is informative for all response variables, including very long IBS tracts close to the selected site (“IBS, close: 30”) and the H12 statistic. Additionally, “LD, mid: 4” represents low LD between sites close to the selected site and sites mid-range from the selected site (likewise for LD “LD, far: 4”). Many low LD pairs could signal a lack of selection, and vice versa. IBS statistics are generally not as helpful for selection, but extremely informative for the population sizes, especially *N*_2_ and *N*_3_, as also observed in the permutation method. Bottlenecks can have a significant impact on IBS tract distributions, so it makes sense that *N*_2_ (the bottleneck size) is the most reliant on IBS statistics. The number of segregating sites *S* is generally quite informative, especially for selection.

It is interesting that the list of notable less-informative statistics is very similar for both permutation and perturbation methods. Overall, roughly the same set of informative statistics were identified by each method, but the precise position of each statistic on the Venn diagram shifted.

### Comparison with ABCtoolbox

Although ABC is not well suited for our scenario of interest and deep learning is a complementary method, we wanted to find a scenario where we could compare the performance of these two methods. To this effect, we restricted the analysis to estimating (continuous) demographic parameters only. We used the popular ABCtoolbox [54], using the same training and testing datasets as for deep learning. For ABC, the training data represents the data simulated under the prior distributions (uniform in our case), and each test dataset was compared with the training data separately. We retained 5% of the training datasets, and used half of these retained datasets for posterior density estimation. Overall, we used 75% of the datasets for training and 25% for testing.

We tested two scenarios, one with the full set of summary statistics (345 total), and the other with a reduced set of summary statistics (100 total). For the reduced set of summary statistics, we chose statistics which seemed to be informative: the number of segregating sites, Tajima’s *D*, the first 15 entries of site frequency spectrum, H1, and the distribution of distances between segregating sites. It is interesting that the informative statistics identified above do not match this set of “intuitively” chosen statistics. The results are shown in Table 8, which suggest that deep learning produces more accurate estimates of the recent population size (*N*_1_) than does ABCtoolbox, whereas they have comparable accuracies for more ancient sizes.

**Table 8:** A comparison between ABCtoolbox and deep learning for demography only. Out of 1000 demographies (160,000 datasets total), 75% were used for training and 25% for testing. In this scenario, deep learning generally outperforms ABCtoolbox, as measured by the relative error: |*N*_est_ − *N*_true_|/*N*_true_. There is generally more improvement using deep learning when the number of statistics is larger.

Dataset	Method	*N*_1_ error	*N*_2_ error	*N*_3_ error
Full summary statistics	ABCtoolbox	0.062	0.043	0.218
	Deep learning	0.044	0.028	0.221
Filtered summary statistics	ABCtoolbox	0.161	0.035	0.311
	Deep learning	0.065	0.055	0.319

### Regularization of the network weights

In deep learning, one hyper-parameter that should be investigated closely is the regularization parameter λ in the cost function, also called the weight decay parameter. (See Methods for details). If λ is set to be too high, large weights will be penalized too much, and interesting features of the data cannot be learned well. But if λ is set too low, the weights tend to display runaway behavior. Due to this balance, a validation procedure is typically used to find the right λ. In our case, the additional runtime of simulating more data and performing more training would be too computationally expensive, but we do provide a small validation study in S3 Fig

### Runtime

In terms of runtime, the majority is spent simulating the data. During training, most of the runtime is spent fine-tuning the deep network, which requires computing the cost function and derivatives many times. To speed up this computation, our deep learning implementation is parallelized across datasets, since each dataset adds to the cost function independently. This significantly improves the training time for deep learning, which can be run in a few days on our simulated dataset (400,000 regions) with a modest number of hidden nodes/layers. Once the training is completed, an arbitrary number of datasets can be tested more or less instantaneously. In contrast, each of the “training” datasets for ABC must be examined for *each* test dataset. This takes several weeks for a dataset of this size (which is why we tested ABC on a subset of the data), although it could be parallelized across the test datasets. We include a runtime comparison on the reduced dataset in Table 9.

**Table 9:** Approximate runtime results. Simulating the data (which is the same for both methods) and computing the summary statistics is the most time consuming part of the process, although it is highly parallelized. ABCtoolbox could also be parallelized, but we did not implement that here. For the “filtered statistics” row, the 345 statistics have been downsampled to 100 for all the datasets.

Task	ABCtoolbox	Deep Learning
Simulating data	370 hrs (10 ∼ 15 cores)	370 hrs (10 ∼ 15 cores)
Computing summary statistics	1800 hrs (1 core)	1800 hrs (1 core)
**Demography only** (1000 × 160 datasets)		
Training and testing (filtered statistics)	114 hrs (1 core)	3.75 hrs (20 cores)
Training and testing (unfiltered)	336 hrs (1 core)	11 hrs (20 cores)
**Demography & selection** (2500 × 160 datasets)		
Training	N/A	74 hrs (20 cores)
Testing	N/A	3 min (1 core)

## Discussion

In this paper, we have sought to demonstrate the potential of deep learning as a new inference framework for population genomic analysis. This investigation is still in its infancy, and there are many directions of future work. One important advantage of deep learning is that it provides a way to distinguish informative summary statistics from uninformative ones. Here we have presented two methods for extracting informative statistics given a trained deep network. Other methods are possible, and it is an open question which one would produce the most accurate results. It would be interesting to down-sample statistics using a variety of methods, then compare the results. Overall, learning more about how various summary statistics relate to parameters would be useful for population genetics going forward.

The prospect of using deep learning to classify regions as neutral or selected is very appealing for subsequent demographic inference. There are other machine learning methods that perform such classification, but they are generally limited to two classes (selected or neutral). One exception is a study in humans [66], which classifies genomic regions as neutral or under positive, negative, or balancing selection. Although their approach does not jointly infer selection and demography, it would be interesting to see their method used on *Drosophila*.

We infer many hard sweeps in African *Drosophila*, which is perhaps expected given their large effective population size. However, when restricting our analysis to selected regions with high confidence, the numbers of hard sweeps and soft sweeps are comparable. It would be interesting to analyze our results in the context of a simulation study by Schrider *et al.* [67], which found that regions classified as soft sweeps are often truly the “shoulders” of hard sweeps. This possibility is worth investigating, as the signatures of soft sweeps and soft shoulders are extremely similar. In our simulations, both hard and soft sweeps are relatively recent, which makes them easier to detect. It would be useful to incorporate datasets with a wider range of selection onset times, which could capture regions of the real data that are currently being classified as neutral. Right now we have 4 different selection classes which are all forms of positive selection. To incorporate background or purifying selection, a 5th class of (weakly) deleterious mutations could be added.

One aspect of the simulations that warrants future study is the effect of the ratio of recombination to mutation rate, *ρ*/*θ*. From running PSMC on 20 haplotypes from Zambia, we inferred *ρ*/*θ* = 1, whereas other studies have found *ρ*/*θ* = 6.8 [59] and *ρ*/*θ* = 10 [58] for a population from Zimbabwe. Both these works also show significant variation in this ratio across different *Drosophila* populations. Andolfatto and Przeworski [60] also find significant variation in this ratio across the genome. As noted by Haddrill *et al.* [59], recent bottlenecks can make this ratio appear lower than it actually is, which is possibly the case for our dataset. As shown in the Supporting Information, misspecification of this ratio can lead to poor selection classification results. In general, assuming a lower recombination rate than reality leads to conservative results, since sites are assumed to be less independent [58–60]. Our takeaway from this analysis is that *ρ*/*θ* should be accurately estimated using a more robust method than PSMC or incorporated as a model parameter, with datasets simulated under a variety of ratios. It would also be interesting to investigate variation in the recombination rate across the genome, which could be incorporated into future simulations.

Deep learning can make efficient use of even a limited number of simulated datasets. In this vein, it would be interesting to use an approach such as ABC MCMC [24] to simulate data, and then use deep learning on these simulated datasets. Alternatively, deep learning could be used to select informative statistics for a subsequent method such as ABC [49]. Blum and François [28] used a single-layer neural network to perform (non-linear) regression to obtain a posterior mean, which they used to adjust the parameters of the posterior sample for a more accurate posterior distribution. It is unclear whether deep networks (where the theory is much more complicated) could be used in the same way, although there is the concept of a residual which could be used for parameter adjustment in an rejection-based framework. Such hybrid approaches could be a fruitful area of further exploration, potentially extending the generality and usefulness of ABC.

We would also like to apply deep learning to a wider variety of scenarios in populations genetics. Population structure and splits would be examples, although these scenarios would most likely require very different sets of summary statistics.

On the computer science side, deep learning has almost exclusively been used for classification, not continuous parameter inference. It would be interesting to see the type of continuous parameter inference presented here used in other fields and applications.

Finally, machine learning methods have been criticized for their “black-box” nature. In some sense they throw away a lot of the coalescent modeling that we know to be realistic, although this is included somewhat in the expert summary statistics of the data. It would be advantageous to somehow combine the strengths of coalescent theory and the strengths of machine learning to create a robust method for population genetic inference.

## Methods

### Deep learning details

In this section we provide the theory behind training deep networks. The notation in this section follows that of [68]. Let ***x***^(*i*)^ be the vector of summary statistics for dataset *i*, and ***y***^(*i*)^ the vector of response variables that dataset *i* was simulated under. If we have *m* such datasets, then together {(***x***^(1)^, ***y***^(1)^), …, (***x***^(*m*)^, ***y***^(*m*)^)} form the training data that will be used to learn the function from summary statistics to response variables. Deep neural networks are a way to express this type of complex, non-linear function. The first layer of the network is the input data, the next layers are the “hidden layers” of the network, and the final layer represents the network’s prediction of the response variable.

#### Cost function for a deep network

**Fig 6:**
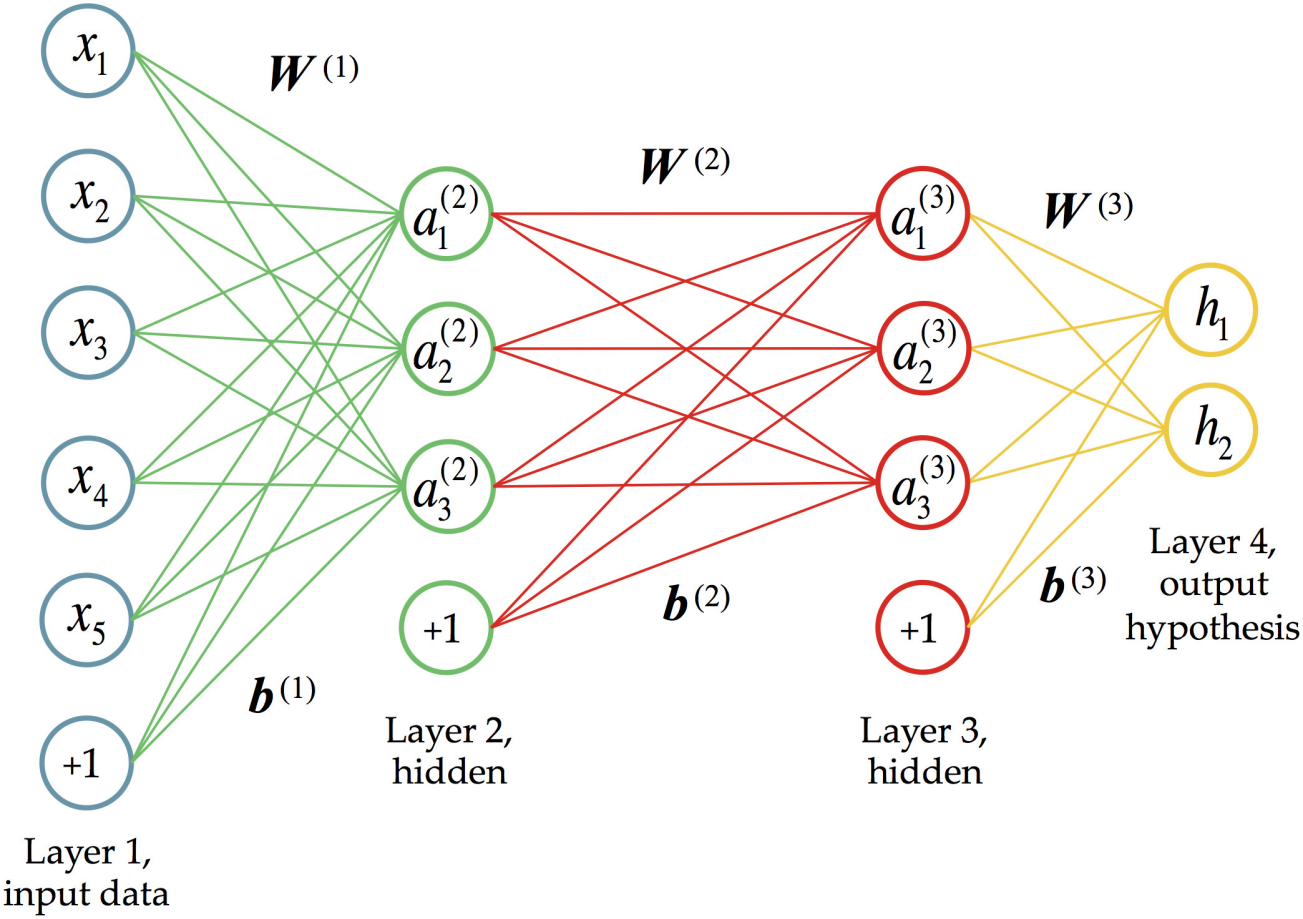
An example of a deep neural network with two hidden layers. The first layer is the input data (each dataset has 5 statistics), and the last layer predicts the 2 response variables. The last node in each input layer (+1) represents the bias term. Here the number of layers *L* = 4, and the number of nodes (computational units) in each layer is *u*_1_ = 5, *u*_2_ = 3, *u*_3_ = 3, and *u*_4_ = 2 (these counts exclude the biases).

An example deep network is shown in Fig 6. The collection of weights between layer *ℓ* and layer *ℓ*+1 is denoted $W^{\left(\ell \right)}=\left(w_{jk}^{\left(\ell \right)}\right)$, where $w_{jk}^{\left(\ell \right)}$ is the weight associated with the connection between node *j* in layer *ℓ* and node *k* in layer *ℓ*+1. The biases for layer *ℓ* is denoted ***b***^(*ℓ*)^. The total number of layers (including the input and output layers) is denoted *L*, and the number of hidden nodes in layer *ℓ* is denoted *u*_*ℓ*_. The main goal is to learn the weights that best describe the function between the inputs and the outputs.

To learn this function, we first describe how the values of the hidden nodes are computed, given a trial weight vector. The value of hidden node *j* in layer *ℓ* ≥ 2 is denoted $a_{j}^{\left(\ell \right)}$, and is defined by

$$a_{j}^{\left(\ell \right)}=f\left(z_{j}^{\left(\ell \right)}\right)\;\text{and}\;z_{j}^{\left(\ell \right)}=W_{j}^{\left(\ell -1\right)}\cdot a^{\left(\ell -1\right)}+b_{j}^{\left(\ell -1\right)},$$ (1)

where $W_{j}^{\left(\ell -1\right)}$ is the *j*^th^ *column* of the weight matrix $W^{\left(\ell -1\right)}$ (i.e., all the weights going into node *j* of layer *ℓ* − 1), $a^{\left(\ell -1\right)}=\left(a_{k}^{\left(\ell -1\right)}\right)$ is a vector of the values of all the nodes in layer *ℓ* − 1 (with $a^{\left(1\right)}=x$, the input vector), and

$$f\left(z\right):=\frac{1}{1+\text{exp}(-z)}$$ (2)

is the *activation function*. Here we use a logistic function, but other functions can be used. Another common activation function is the hyperbolic tangent function.

Hence, given the input data and a set of weights, we can *feed forward* to learn the values of all hidden nodes, and a prediction of the response variables. These predictions are usually denoted by ***h***_***W*, *b***_(***x***^(*i*)^) for our *hypothesis* for dataset *i*, based on all the weights ***W*** = (***W***^(1)^, …,***W***^(*L*-1)^) and biases ***b*** = (***b***^(1)^, …,***b***^(*L*-1)^). We will discuss different ways to compute the hypothesis function later on. To find the best weights, we define a loss function based on the square of the *l*_2_-norm between this hypothesis and the true response variables. This loss function is given by

$$J\left(W,b\right)=\frac{1}{m}\sum_{i=1}^{m}\frac{1}{2}\|h_{W,b}\left(x^{\left(i\right)}\right)-y^{\left(i\right)}{\|}^{2}.$$ (3)

The goal of deep learning is to find the weights (***W*** and ***b***) that minimize this loss function. To efficiently find these optimal weights, we can use *backpropagation* to find the gradient. The intuition behind this approach is that once we have found the hypothesis, we then want to see how much each of the weights contributed to any differences between the hypothesis and the truth. Therefore we start at the last hidden layer, see how much each of those weights contributed, then work our way backwards, using the gradient of the previous layer to compute the gradient of the next layer. For this procedure we need to compute the partial derivatives of the cost function with respect to each weight.

Consider a single training example ***x*** with associated output ***y***. The cost for this dataset is a term in Eq (3) and is denoted by

$$J\left(W,b,x,y\right):=\frac{1}{2}\|h_{W,b}\left(x\right)-y{\|}^{2}.$$

Then, define

$$\delta_{j}^{\left(\ell \right)}:=\frac{\partial J(W,b,x,y)}{\partial z_{j}^{\left(\ell \right)}},$$

where $z_{j}^{\left(\ell \right)}$, defined in Eq (1), is the input to the activation function for node *j* in layer *ℓ*. We first consider $\delta_{j}^{\left(L\right)}$ for the final layer. Noting that the *j*^th^ entry of ***h***_***W*, *b***_(***x***) is $f(z_{j}^{\left(L\right)})$, we get

$$\delta_{j}^{\left(L\right)}=\left[f\left(z_{j}^{\left(L\right)}\right)-y_{j}\right]f^{\prime }\left(z_{j}^{\left(L\right)}\right).$$

Based on this initialization, we can recursively compute all the *δ* variables:

$$\delta_{j}^{\left(\ell \right)}= [\sum_{k=1}^{u_{\ell +1}}w_{jk}^{\left(\ell \right)}\delta_{k}^{\left(\ell +1\right)}]f^{\prime }\left(z_{j}^{\left(\ell \right)}\right).$$

Now we can use the *δ* variables to recursively compute the partial derivatives for one dataset:

$$\begin{array}{lll} \frac{\partial J\left(W,b,x,y\right)}{\partial w_{jk}^{\left(\ell \right)}} & = & a_{j}^{\left(\ell \right)}\delta_{k}^{\left(\ell +1\right)},\\ \frac{\partial J\left(W,b,x,y\right)}{\partial b_{k}^{\left(\ell \right)}} & = & \delta_{k}^{\left(\ell +1\right)}. \end{array}$$

Finally, putting all the datasets together we get

$$\frac{\partial J(W,b)}{\partial w_{jk}^{\left(\ell \right)}}=\sum_{i=1}^{m}\frac{\partial J(W,b,x^{\left(i\right)},y^{\left(i\right)})}{\partial w_{jk}^{\left(\ell \right)}}\;\text{and}\;\frac{\partial J(W,b)}{\partial b_{k}^{\left(\ell \right)}}=\sum_{i=1}^{m}\frac{\partial J(W,b,x^{\left(i\right)},y^{\left(i\right)})}{\partial b_{k}^{\left(\ell \right)}}.$$

Since we can compute the derivatives using this backpropagation algorithm, we can use the LBFGS optimization routine (as implemented in [69]) to find the weights that minimize the cost function.

#### Unsupervised pretraining using autoencoders

It is possible to train a deep network by attempting to minimize the cost function described above directly, but in practice, this proved difficult due to the high-dimensionality and non-convexity of the optimization problem. Initializing the weights randomly before training resulted in poor local minima. Hinton and Salakhutdinov [37] sought to initialize the weights in a more informed way, using an unsupervised pretraining routine. Unsupervised training ignores the output (often called the “labels”) and attempts to learn as much as possible about the structure of the data on its own. PCA is an example of unsupervised learning. In Hinton and Salakhutdinov, the unsupervised pretraining step uses an *autoencoder* to try to learn the best function from the data to itself, after it has gone through a dimensionality reduction step (this can be thought of as trying to compress the data, then reconstruct it with minimal loss). Autoencoding provides a way to initialize the weights of a deep network that will ideally be close to optimal for the supervised learning step as well. See S4 Fig for a diagram of an autoencoder.

Training an autoencoder is an optimization procedure in itself. As before, let ***W***^(1)^ be the vector of weights connecting the input ***x*** to the hidden layer ***a***, and ***x***^(2)^ be the vector of weights connecting ***a*** to the output layer $\hat{x}$, which in this case should be as close as possible to the original input data. We again typically use the logistic function shown in Eq (2) as our activation function *f*, so we can compute the output using:

$$a_{j}=f\left(W_{j}^{\left(1\right)}\cdot x+b_{j}^{\left(1\right)}\right)\;\text{and}\;{\hat{x}}_{k}=f\left(W_{k}^{\left(2\right)}\cdot a+b_{k}^{\left(2\right)}\right).$$

If a linear activation function is used instead of a logistic function, the hidden layer becomes the principle components of the data. This makes dimensionality reduction with an autoencoder similar in spirit to PCA, which has been used frequently in genetic analysis (see [70] for an example). However, the non-linear nature of an autoencoder has been shown to reconstruct complex data more accurately than PCA. Using backpropagation as we did before, we can minimize the following autoencoder cost function using all *m* input datasets:

$$A\left(W,b\right)=\frac{1}{m}\sum_{i=1}^{m}\frac{1}{2}\|{\hat{x}}^{\left(i\right)}-x^{\left(i\right)}{\|}^{2}.$$

The resulting weights W*^(1)^ and b*^(1)^ will then be used to initialize the weights between the first and second layers of our deep network. The weights W*^(2)^> and b*^(2)^ are discarded. To initialize the rest of the weights, we can repeat the autoencoder procedure, but this time we will use the hidden layer $a^{*}=\left(a_{j}^{*}\right)$, where $a_{j}^{*}=f\left(W_{j}^{*(1)}\cdot x+b_{j}^{*(1)}\right)$, as our input data, and feed it through the next hidden layer. In this way we can use “stacked” autoencoders to initialize all the weights of the deep network. Finally, the supervised training procedure described in the previous section can be used to *fine-tune* the weights to obtain the best function from the inputs to the response variables.

When the number of hidden nodes is large, we would like to constrain an autoencoder such that only a fraction of the hidden nodes are “firing” at any given time. This corresponds to the idea that only a subset of the neurons in our brains are firing at once, depending on the input stimulus. To create a similar phenomenon for an autoencoder, we create a *sparsity* constraint that ensures the activation of most of the nodes is close to 0, and the activation of a small fraction, *ρ*, of nodes is close to 1. Let ${\hat{\rho }}_{j}$ be the average activation of the hidden node *j*:

$${\hat{\rho }}_{j}=\frac{1}{m}\sum_{i=1}^{m}a_{j}\left(x^{\left(i\right)}\right),$$

where $a_{j}\left(x^{\left(i\right)}\right)$ is the value of the *j*^th^ hidden node when activated with dataset ***x***^(*i*)^. To ensure sparsity, we would like ${\hat{\rho }}_{j}$ to be close to *ρ*, our desired fraction of active nodes. This can be accomplished by minimizing the KL divergence:

$$\sum_{j=1}^{u_{2}}\text{KL}\left(\rho \|{\hat{\rho }}_{j}\right)=\sum_{j=1}^{u_{2}} [\rho \log\frac{\rho }{{\hat{\rho }}_{j}}+\left(1-\rho \right)\log\frac{1-\rho }{1-{\hat{\rho }}_{j}}],$$

where *u*_2_ is the number of nodes in the hidden layer of the autoencoder. We multiply this term by a sparsity weight *β*.

In addition, a regularization term is included, which prevents the magnitude of the weights from becoming too large. To accomplish this, we add a penalty to the cost function that is the sum of the squares of all weights (excluding the biases), weighted by a well-chosen constant λ, which is often called the *weight decay parameter*. Including both sparsity and regularization, our final autoencoder cost becomes:

$$A_{\lambda }\left(W,b\right)=\frac{1}{m}\sum_{i=1}^{m}\frac{1}{2}\|{\hat{x}}^{\left(i\right)}-x^{\left(i\right)}{\|}^{2}+\beta \sum_{j=1}^{u_{2}}\text{KL}\left(\rho \|{\hat{\rho }}_{j}\right)+\frac{\lambda }{2}\sum_{\ell =1}^{2}\sum_{j=1}^{u_{\ell }}\sum_{k=1}^{u_{\ell +1}}{ [(w_{jk}^{\left(\ell \right)}]}^{2}.$$

We also regularize the weights on the last layer during fine-tuning, so our deep learning cost function becomes:

$$J_{\lambda }\left(W,b\right)=\frac{1}{m}\sum_{i=1}^{m}\frac{1}{2}\|h_{W,b}\left(x^{\left(i\right)}\right)-y^{\left(i\right)}{\|}^{2}+\frac{\lambda }{2}\sum_{j=1}^{u_{L-1}}\sum_{k=1}^{u_{L}}{ [w_{jk}^{\left(L-1\right)}]}^{2}.$$

#### The final layer: Continuous variable vs. classification

In population genetics, often we want to estimate continuous response variables. To compute our hypothesis for a response variable, based on a set of weights, we could use a logistic activation function, Eq (2), as we did for the other layers. However, the logistic function is more suitable for binary classification. Instead, we use a linear activation function, so in the case of a single response variable, our hypothesis for dataset *i* becomes

$$h_{W,b}^{\text{linear}}\left(x^{\left(i\right)}\right)={\left[a^{\left(L-1\right)}\left(x^{\left(i\right)}\right)\right]}^{T}W^{\left(L-1\right)}+b^{\left(L-1\right)}.$$

In other words, it is the dot product of the activations of the final hidden layer and the weights that connect the final hidden layer to the response variables.

For such classification results, if we had two classes, we could use logistic regression to find the probability a dataset was assigned to each class. With more than two classes, we can extend this concept and use *softmax regression* to assign a probability to each class. If we have *K* classes labeled 1, …, *K*, we can define our hypothesis as follows

$$\begin{array}{lll} h_{W,b}^{\text{softmax}}\left(x^{\left(i\right)}\right) & = & \left[\begin{array}{c} p\left(y^{\left(i\right)}=2|x^{\left(i\right)};W,b\right)\\ p\left(y^{\left(i\right)}=2|x^{\left(i\right)};W,b\right)\\ \vdots \\ p\left(y^{\left(i\right)}=K|x^{\left(i\right)};W,b\right) \end{array}\right]\\ & = & \frac{1}{Z}\left[\begin{array}{c} \text{exp}\left\{W_{1}^{\left(L-1\right)}\cdot a^{\left(L-1\right)}\left(x^{\left(i\right)}\right)+b_{1}^{\left(L-1\right)}\right\}\\ \text{exp}\left\{W_{2}^{\left(L-1\right)}\cdot a^{\left(L-1\right)}\left(x^{\left(i\right)}\right)+b_{2}^{\left(L-1\right)}\right\}\\ \vdots \\ \text{exp}\left\{W_{K}^{\left(L-1\right)}\cdot a^{\left(L-1\right)}\left(x^{\left(i\right)}\right)+b_{K}^{\left(L-1\right)}\right\} \end{array}\right], \end{array}$$

where *Z* is the sum of all the entries, so that our probabilities sum to 1. Using this formulation, we can define our classification cost function:

$$J_{\lambda }^{\text{softmax}}\left(W,b\right)=-\frac{1}{m}\sum_{i=1}^{m}\sum_{j=1}^{K}1\left\{y^{\left(i\right)}=j\right\}\log p\left(y^{\left(i\right)}=j|x^{\left(i\right)};W,b\right)+\frac{\lambda }{2}\sum_{s=1}^{u_{L-1}}\sum_{t=1}^{u_{L}}{ [W_{st}^{\left(L-1\right)}]}^{2}.$$

Intuitively, we can think about this cost function as making the log probability of the correct class as close to 0 as possible.

### Transforming input data into summary statistics

**Fig 7:**
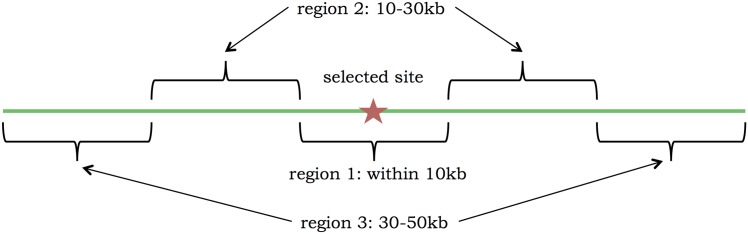
Regions used for computing the statistics, which are based off of Peter *et al.* [23]. Note that the selected site was chosen randomly within region 1.

For many deep learning applications, the raw data can be used directly (the pixels of an image, for example). Unfortunately, we currently cannot input raw genomic data into a deep learning method. Similarly to ABC, we need to transform the data into summary statistics that are potentially informative about the parameters of interest. Unlike ABC, however, deep learning should not be negatively affected by correlated or uninformative summary statistics. Thus we sought to include a large number of potentially informative summary statistics of the data. To account for the impact of selection, we divided each 100 kb region into three smaller regions: 1) close to the selected site (40–60 kb), 2) mid-range from the selected site (20–40 kb and 60–80 kb), and 3) far from the selected site (0–20 kb and 80–100 kb). These regions are based off of the simulation scenario in Peter *et al.* [23], and shown more explicitly in Fig 7. Within each of these three regions, we calculated the following statistics, except where noted.

For all the statistics described below, *n* is the haploid sample size. In the case of simulated data, *n* = 100. For the real data, we had 197 samples; within each 100 kb region we sorted the samples by missing data, then retained the 100 most complete samples, except where noted.

1. Number of segregating sites within each smaller region, *S*. Since all of the statistics must be in [0, 1], we normalized by *S*_max_ = 5000 (any *S* > *S*_max_ was truncated): 3 statistics.
2. Tajima’s *D* statistic [71], computed as follows
  $$D=\frac{\pi -S/a_{1}}{\sqrt{\hat{\text{Var}}\left(\pi -S/a_{1}\right)}},$$
  where *π* is the average number of pairwise differences between two samples, and $a_{1}=\sum_{i=1}^{n-1}1/i$. We normalized by *D*_min_ = −3.0 and *D*_max_ = 3.0, again truncating the rare statistic outside this range: 3 statistics.
3. Folded site frequency spectrum (SFS): *η*_*i*_ is the number of segregating sites where the minor allele occurs *i* times out of *n* samples, for *i* = 1, 2, …, ⌊*n*/2⌋. For the real data, for each segregating site, enough samples were included to obtain 100 with non-missing data. If that was not possible for a given site, the site was not included. To normalize the SFS, we divided each *η*_*i*_ by the sum of the entries, which gives us the probability of observing *i* minor alleles, given the site was segregating: 50 ⋅ 3 = 150 statistics.
4. Length distribution between segregating sites: let *B*_*k*_ be the number of bases between the *k* and *k*+1 segregating sites. To compute the distribution of these lengths, we define *J* bins and count the number of lengths that fall into each bin:
  $$d_{j}^{\text{BET}}=\frac{|k\in \left\{1,2,\cdots ,S-1\right\}:B_{k}\in \text{bin}\;j|}{S}.$$
  We choose *J* = 16 equally spaced bins, the first starting at 0 and the last starting at 300: 16 ⋅ 3 = 48 statistics.
5. Identity-by-state (IBS) tract length distribution: for each pair of samples, and IBS tract is a contiguous genomic region where the samples are identical at every base (delimited by bases where they differ). For all pairs, let *L* be the set of IBS tract lengths. In a similar fashion to the length distribution statistics, we define *M* bins, and count the number of IBS tract lengths that fall into each bin:
  $$d_{m}^{\text{IBS}}=\frac{\left|\ell \in L:\ell \in \text{bin}\;m\right|}{\left|L\right|}.$$
  We choose *M* = 30 equally spaced bins, the first starting at 0 and the last starting at 5000: 30 ⋅ 3 = 90 statistics.
6. Linkage disequilibrium (LD) distribution: LD is a measure of the correlation between two segregating sites. For example, if there was no recombination between two sites, their alleles would be highly correlated and LD would be high in magnitude. If there was infinite recombination between two sites, they would be independent and have LD close to 0. For two loci, let *A*, *a* be the alleles for the first site, and *B*, *b* be the alleles for the second site. Let *p*_*A*_ be the frequency of allele *A*, *p*_*B*_ be the frequency of allele *B*, and *p*_*AB*_ be the frequency of haplotype *AB*. Then the linkage disequilibrium is computed by
  $$D_{AB}=p_{AB}-p_{A}p_{B}.$$
  Here we compute the LD between pairs of sites, where one site is in the “selected” region, and the other is in each of the three regions (including the selected region). Then we create an LD distribution similar to the IBS distribution above, using 16 bins, with the first bin ending at −0.05, and the last bin starting at 0.2: 16 ⋅ 3 = 48 statistics.
7. H1, H12, and H2 statistics, as described in Garud *et al.* [72]. These statistics help to distinguish between hard and soft sweeps, and are calculated in the selected (middle) region only: 3 statistics.

This gives us a total of 345 statistics.

### Deep learning for demography and selection

#### Deep learning model

To modify our deep learning method to accommodate this type of inference problem, during training we have an outer-loop that changes the demography as necessary, and an inner loop that accounts for differences in selection for each region. During testing, we compare three different methods for predicting the effective population sizes: (1) *Average stat*: we average the statistics for each region within the dataset (corresponding to the same demography), then run this average through the trained network. (2) *Final*: we run the statistics for each region through the trained network separately, then average the results. (3) *Neutral regions*: we use the same method as (2), but instead of using all the regions, we only use the ones we predict as being neutral. We also include a range between the 2.5th and 97.5th quantiles. To predict the selection class, we obtain a probability distribution over the classes for each region, then select the class with the highest probability.

**Fig 8:**
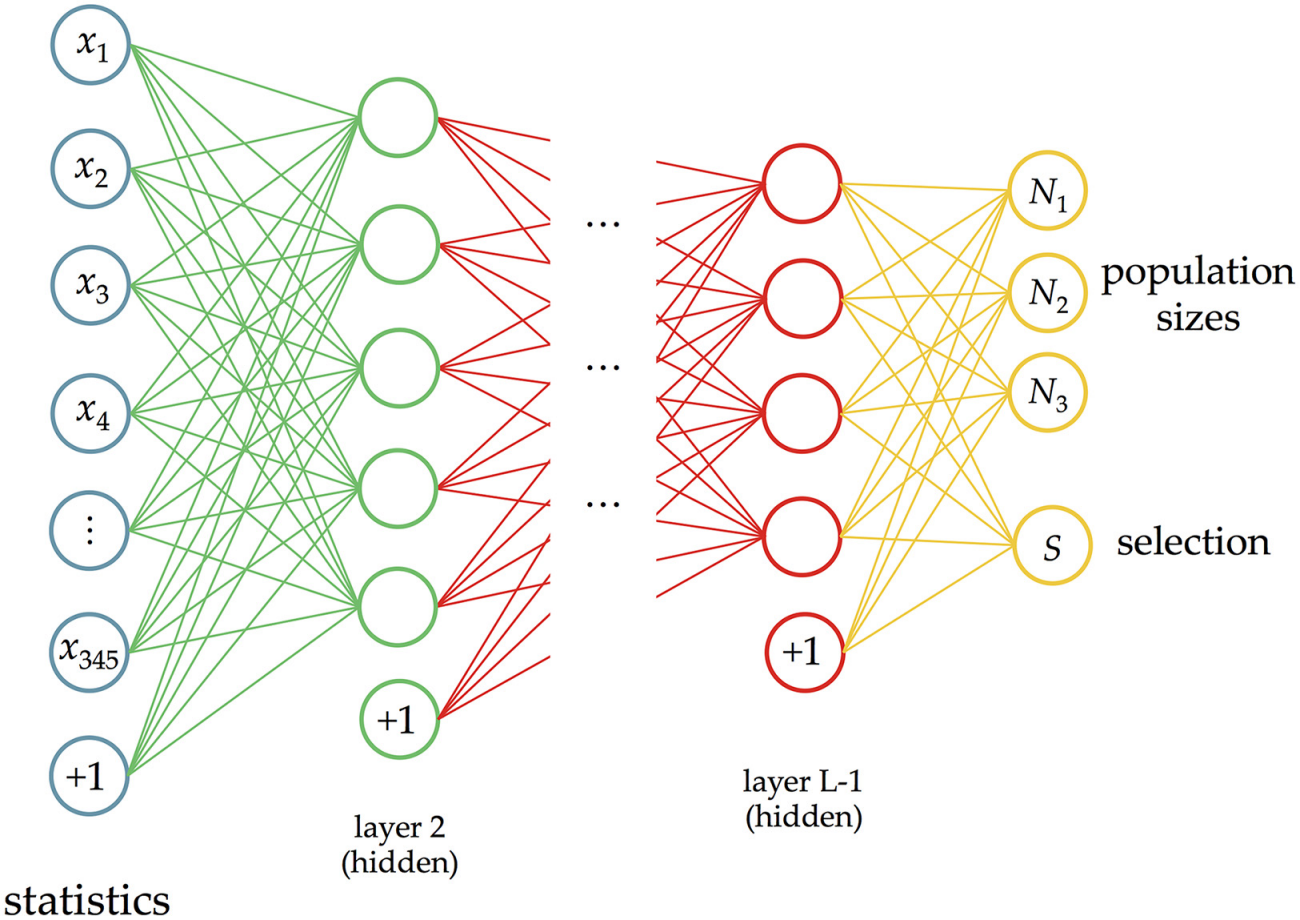
Our deep learning framework for effective population size changes and selection.

One final complication is that we estimate continuous response variables for the population sizes, but consider selection to be a discrete response variable. This involves a linear activation function for the population sizes and a softmax classifier for selection. A diagram of our deep learning method is shown in Fig 8.

#### Analysis of informative statistics

One advantage of deep learning is that the weights of the optimal network give us an interpretable link between the summary statistics and the output variables. However, from these weights, it is not immediately obvious which statistics are the most informative or “best” for a given output variable. If we only wanted to use only a small subset of the statistics, which ones would give us the best results? To answer this question, we use both a permutation testing approach and a perturbation method described in S1 Algorithm.

### Software availability

An open-source software package (called evoNet) that implements deep learning algorithms for population genetic inference is available at https://sourceforge.net/projects/evonet.

## Supporting Information

S1 Table Results for very different training and testing data. In this scenario, the testing data is simulated with a recombination rate that is 4 times higher than that of the training data. The effective population size results (top table) are still generally accurate, but selection (bottom table) is harder to predict. Neutral regions are predicted correctly, but soft sweeps are also often classified as neutral. The classifier has difficulty distinguishing between hard sweeps and balancing selection. The top table should be compared to Table 1 and the bottom table should be compared to Table 3.
(TIFF)

S2 Table Results for very different training and testing data. In this scenario, the testing data is simulated with bottleneck parameters that are more severe than the training data. This has a slight negative impact on the population size results (largely on the most recent size which was outside the training range), but has little effect on the selection results. The overall percentage of misclassified regions is 7.8%. The top table should be compared to Table 1 and the bottom table should be compared to Table 3.
(TIFF)

S3 Table Classification results for all windows, along with which genes are found in each window. (XLS)

S4 Table High-confidence windows, along with which genes are found in each window. (XLS)

S1 Fig An example of a classical neural network. The single hidden layer serves to learn informative combinations of the inputs, remove correlations, and typically reduce the dimension of the data. After the optimal weight on each connecting arrow is learned through labeled training data, unlabeled data can be fed through the network to estimate the response variables.
(TIFF)

S2 Fig A Venn diagram of most informative statistics for each output variable (*N*_1_, *N*_2_, *N*_3_, and selection), using the perturbation method. For each variable, the top 25 statistics were chosen, according to the procedure in S1 Algorithm. The Venn diagram captures statistics common to each subset of output variables, with notable less informative statistics shown in the lower right. Close, mid, and far represent the genomic region where the statistic was calculated. The numbers after each colon refer to the position of the statistic within its distribution or order. For the SFS statistics, it is number of minor alleles. For each region, there are 50 SFS statistics, 16 BET statistics (distribution between segregating sites), 30 IBS statistics, and 16 LD statistics.
(TIFF)

S3 FigValidation procedure to find the optimal weight decay parameter]Validation procedure for a network with two hidden layers of size 8 and 4.The x-axis shows increasing values of λ, and the y-axis shows the error on the validation dataset. The curve shows a characteristic shape with low and high λ producing poorer results than an intermediate value. For these hidden layers sizes and this dataset, $\hat{\lambda }=0.0001$ was optimal.(TIFF)

S4 FigExample of an autoencoder]An example of an autoencoder.The input data (**x**) is projected into a (usually) lower dimension (**a**), then reconstructed ($\hat{x}$). The weights of an autoencoder are optimized such that the difference between the reconstructed data and the original data is minimal.(TIFF)

S1 Algorithm Method for finding the most informative statistics for each response variable. (TIFF)
