# Supplementary figures and images for: Deep Learning for Population Genetic Inference
Source: PLoS Comput Biol. 2016 Mar 28;12(3):e1004845. doi: 10.1371/journal.pcbi.1004845 (PMC4809617; doi:10.1371/journal.pcbi.1004845)

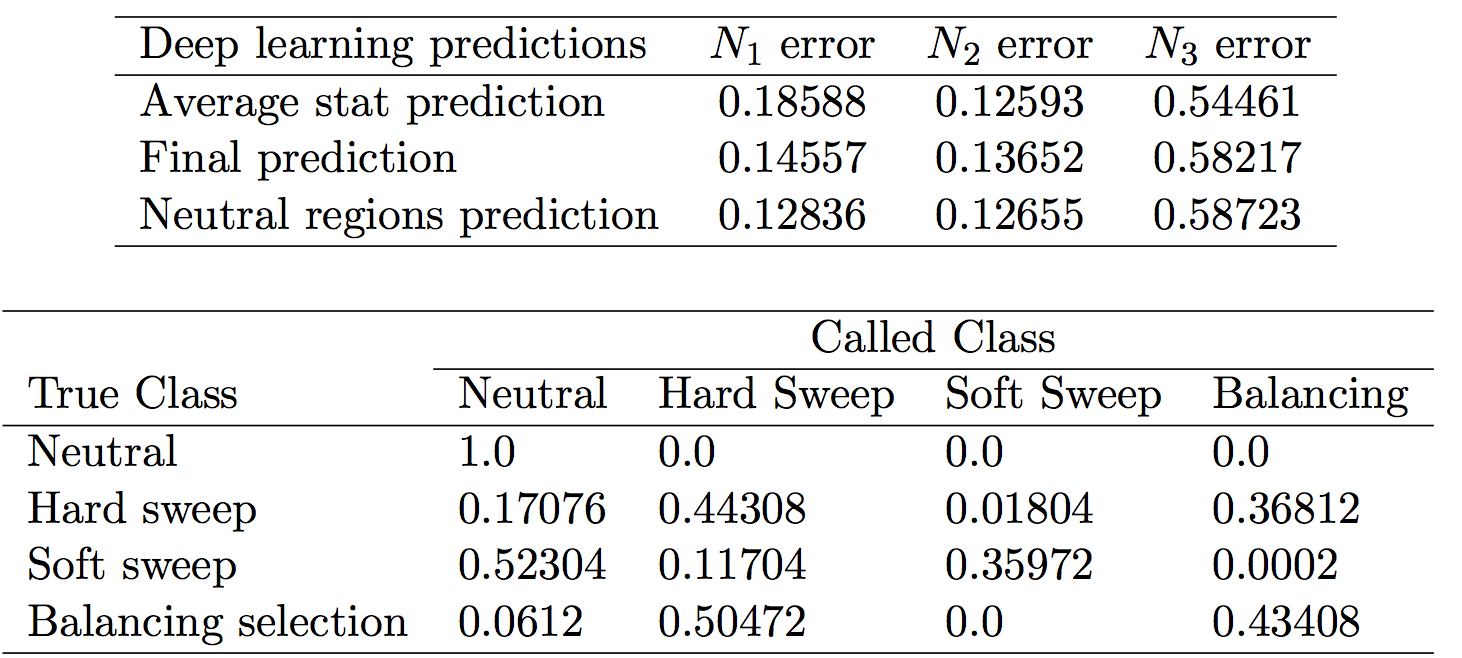

Supplement: S1 Table — In this scenario, the testing data is simulated with a recombination rate that is 4 times higher than that of the training data. The effective population size results (top table) are still generally accurate, but selection (bottom table) is harder to predict. Neutral regions are predicted correctly, but soft sweeps are also often classified as neutral. The classifier has difficulty distinguishing between hard sweeps and balancing selection. The top table should be compared to Table 1 and the bottom table should be compared to Table 3. (TIFF) [file pcbi.1004845.s001.tiff]

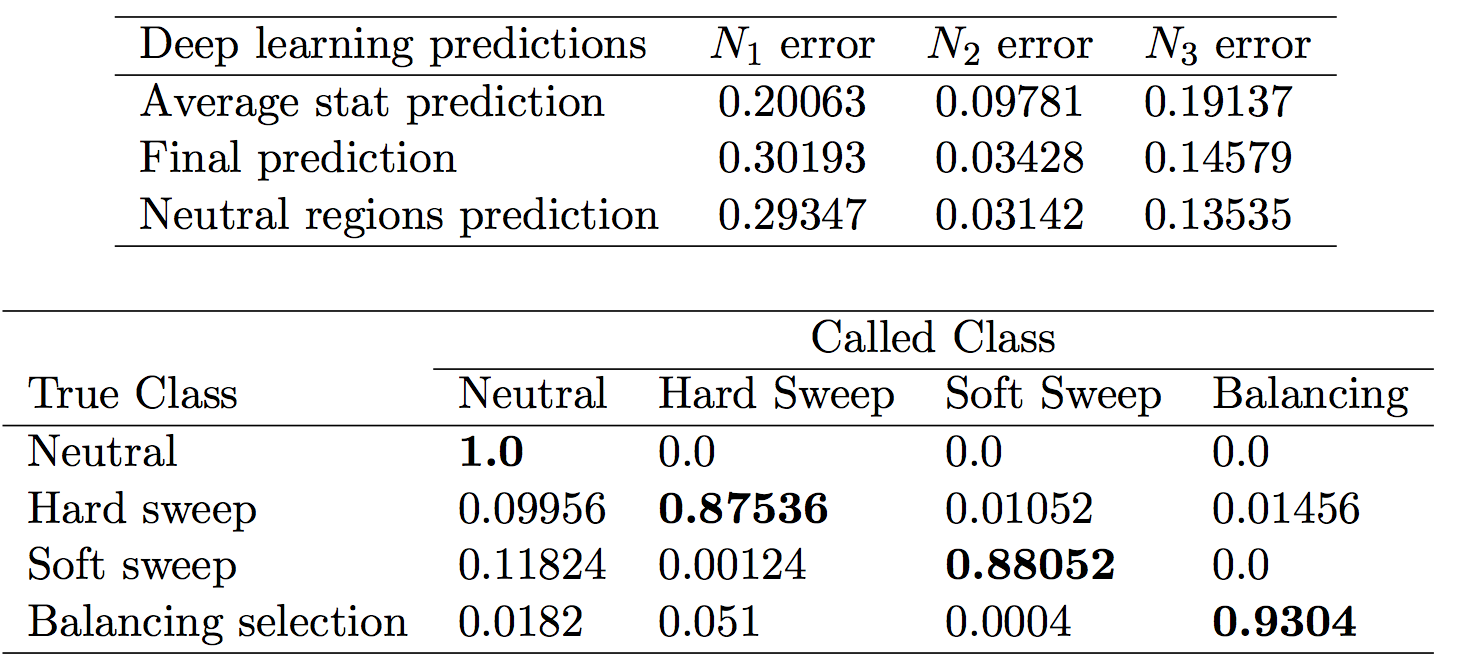

Supplement: S2 Table — In this scenario, the testing data is simulated with bottleneck parameters that are more severe than the training data. This has a slight negative impact on the population size results (largely on the most recent size which was outside the training range), but has little effect on the selection results. The overall percentage of misclassified regions is 7.8%. The top table should be compared to Table 1 and the bottom table should be compared to Table 3. (TIFF) [file pcbi.1004845.s002.tiff]

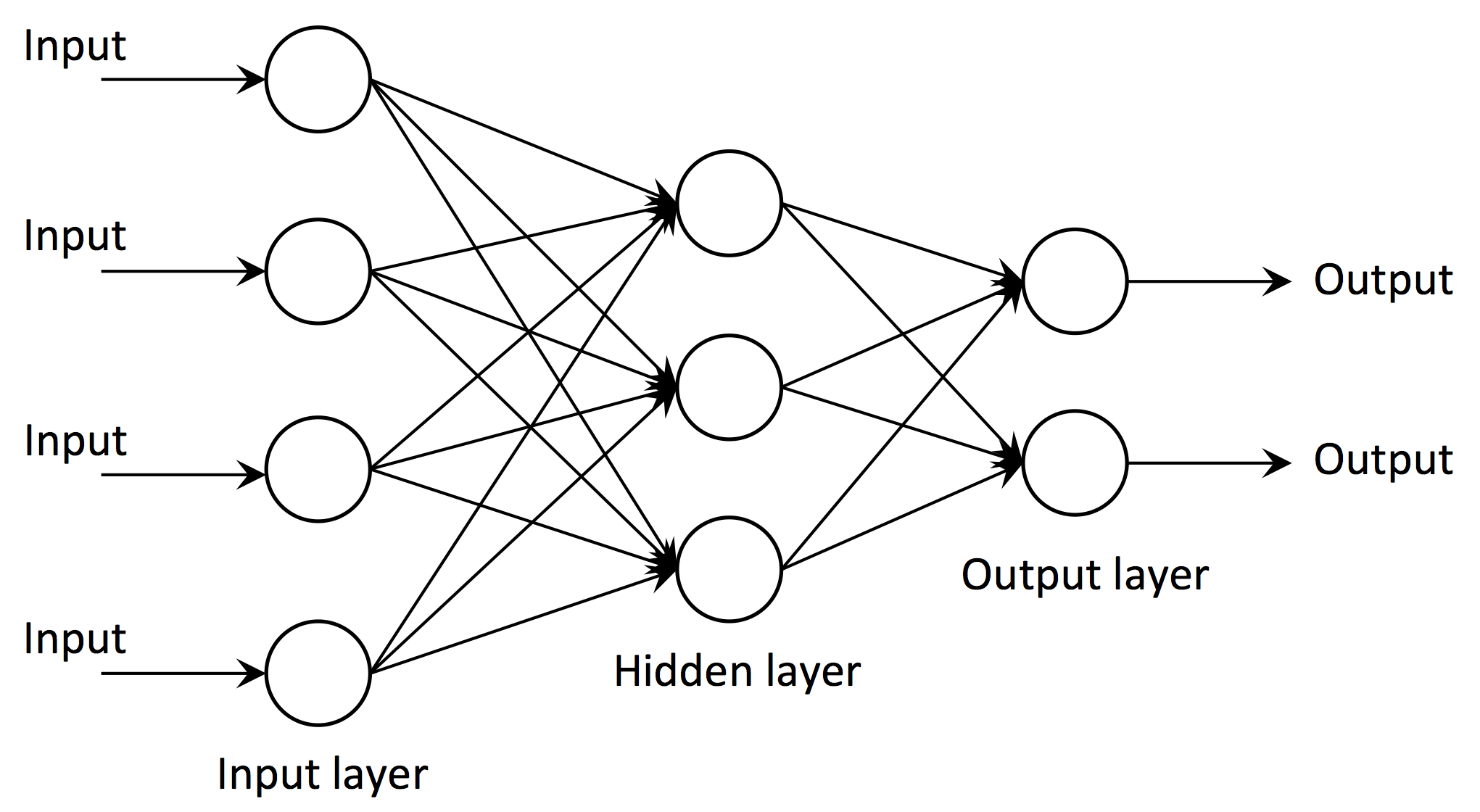

Supplement: S1 Fig — The single hidden layer serves to learn informative combinations of the inputs, remove correlations, and typically reduce the dimension of the data. After the optimal weight on each connecting arrow is learned through labeled training data, unlabeled data can be fed through the network to estimate the response variables. (TIFF) [file pcbi.1004845.s005.tiff]

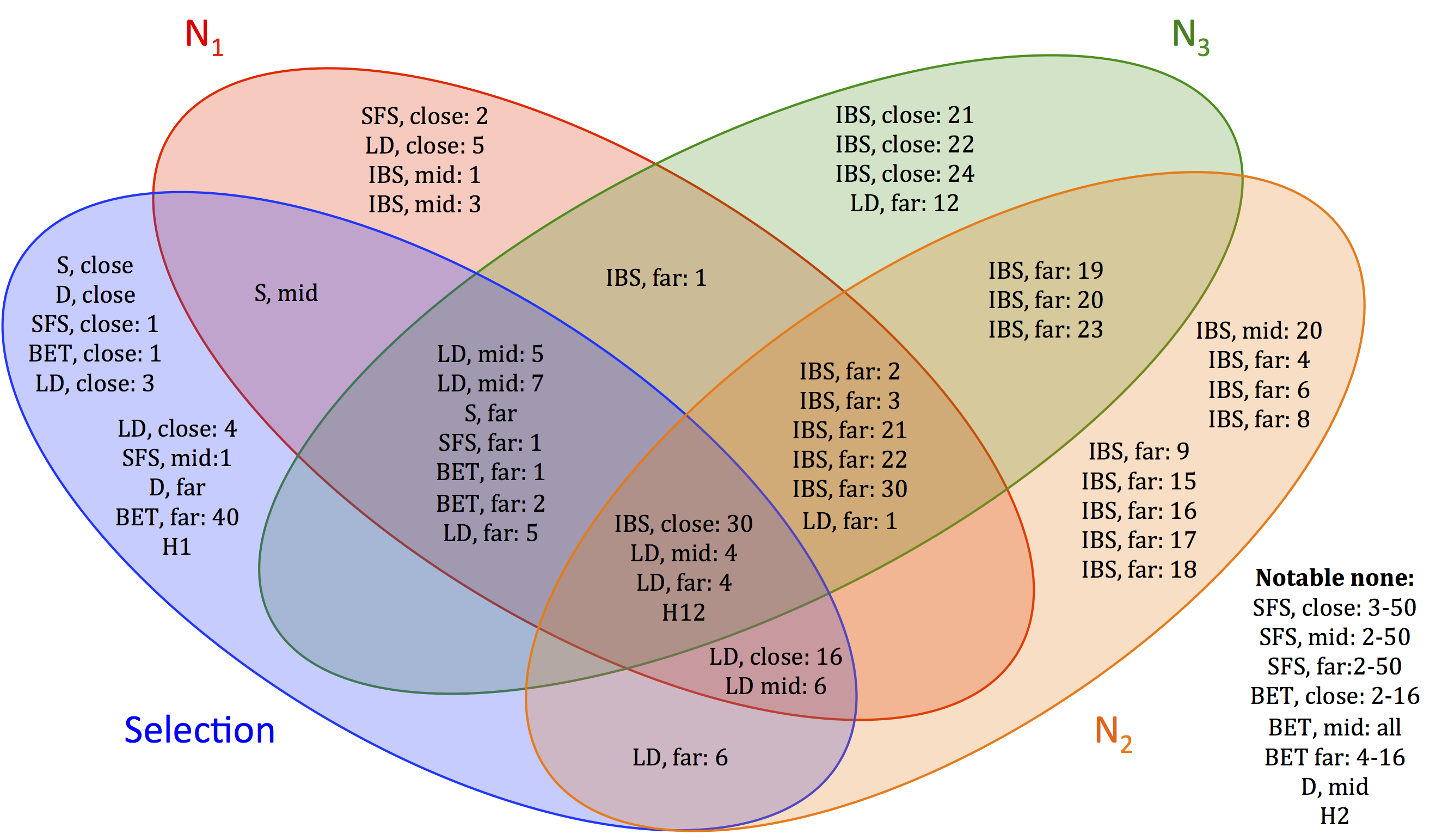

Supplement: S2 Fig — For each variable, the top 25 statistics were chosen, according to the procedure in S1 Algorithm. The Venn diagram captures statistics common to each subset of output variables, with notable less informative statistics shown in the lower right. Close, mid, and far represent the genomic region where the statistic was calculated. The numbers after each colon refer to the position of the statistic within its distribution or order. For the SFS statistics, it is number of minor alleles. For each region, there are 50 SFS statistics, 16 BET statistics (distribution between segregating sites), 30 IBS statistics, and 16 LD statistics. (TIFF) [file pcbi.1004845.s006.tiff]

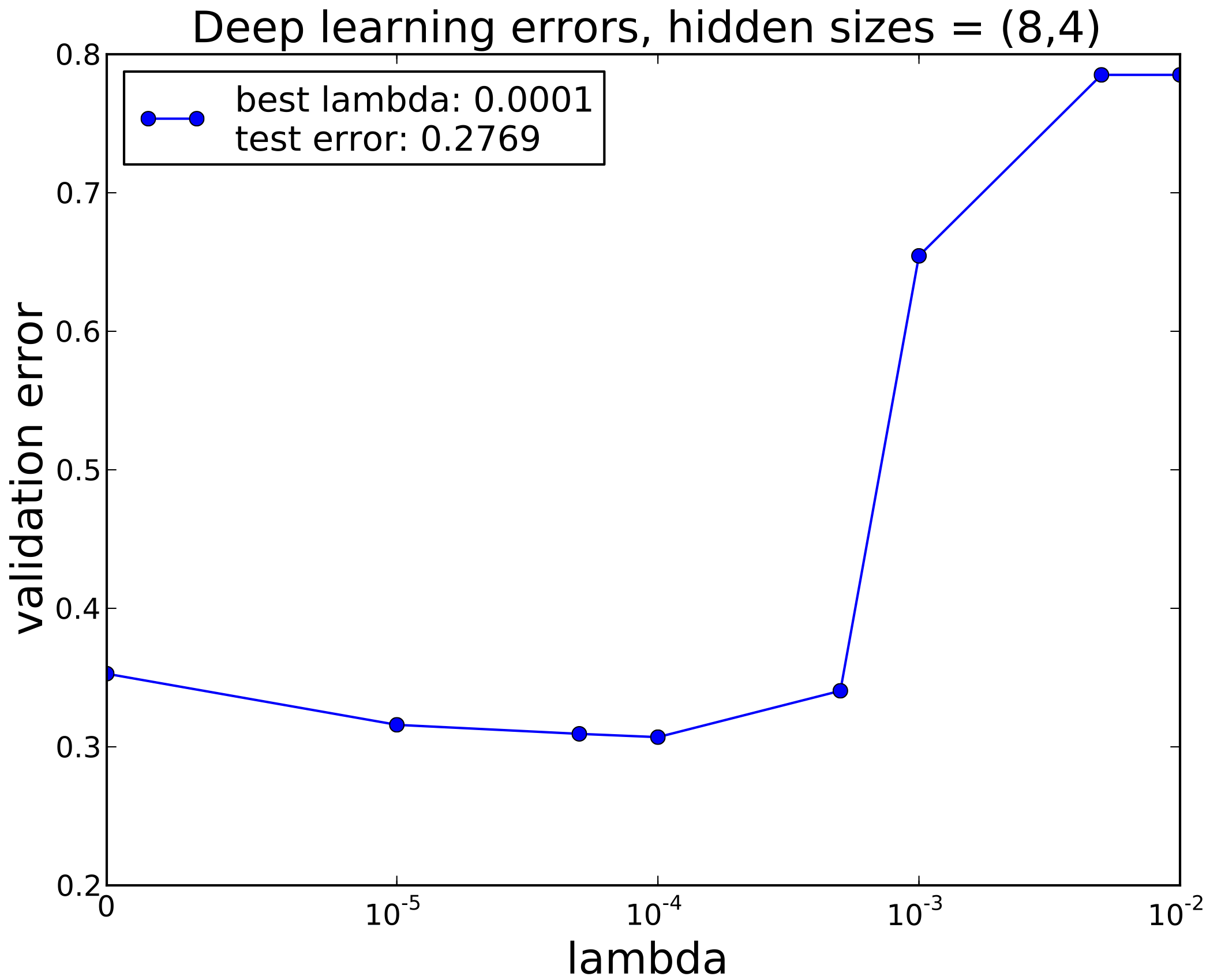

Supplement: S3 Fig — The x-axis shows increasing values of λ, and the y-axis shows the error on the validation dataset. The curve shows a characteristic shape with low and high λ producing poorer results than an intermediate value. For these hidden layers sizes and this dataset, λ^=0.0001 was optimal. (TIFF) [file pcbi.1004845.s007.tiff]

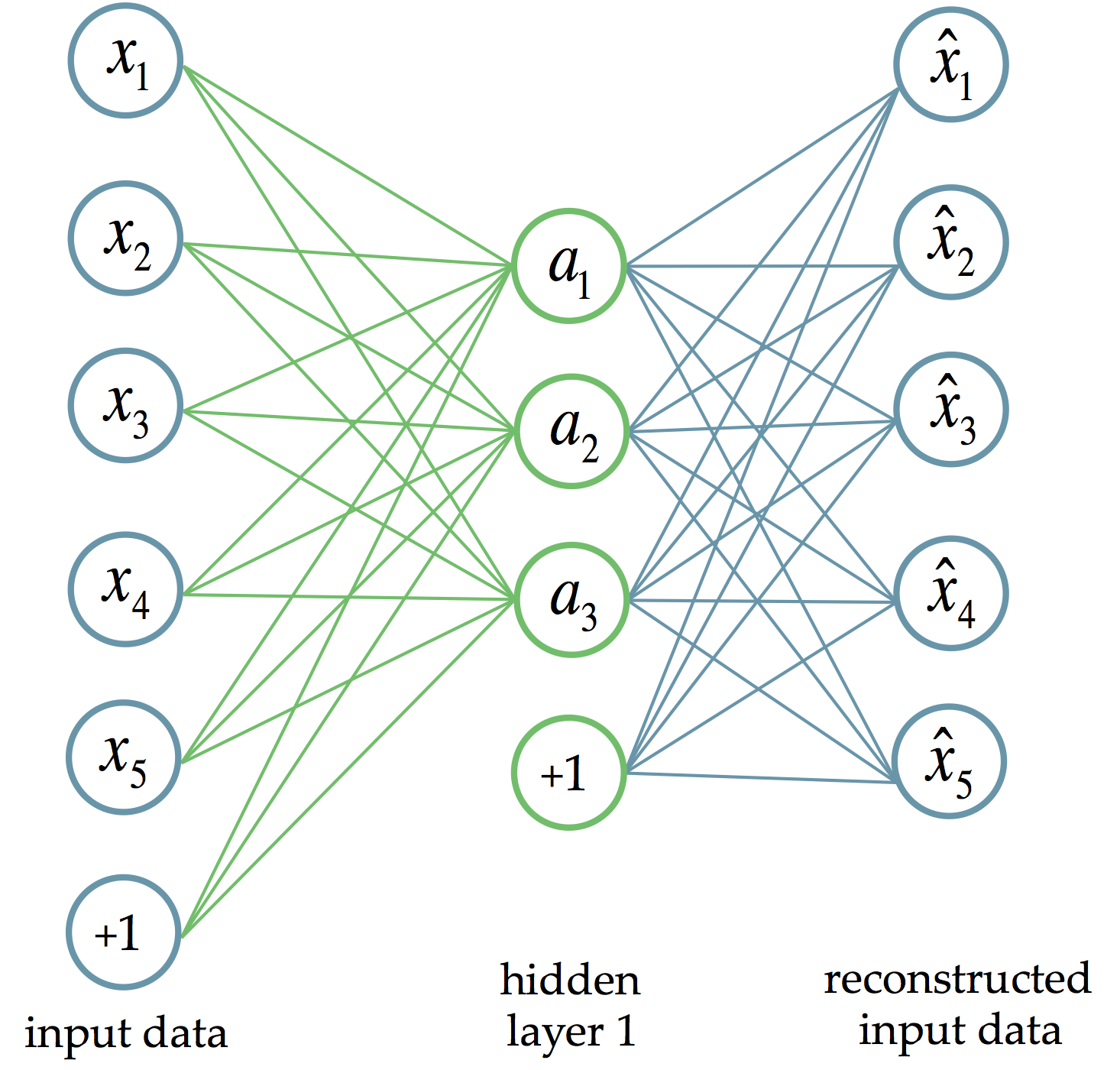

Supplement: S4 Fig — The input data (x) is projected into a (usually) lower dimension (a), then reconstructed (x^). The weights of an autoencoder are optimized such that the difference between the reconstructed data and the original data is minimal. (TIFF) [file pcbi.1004845.s008.tiff]

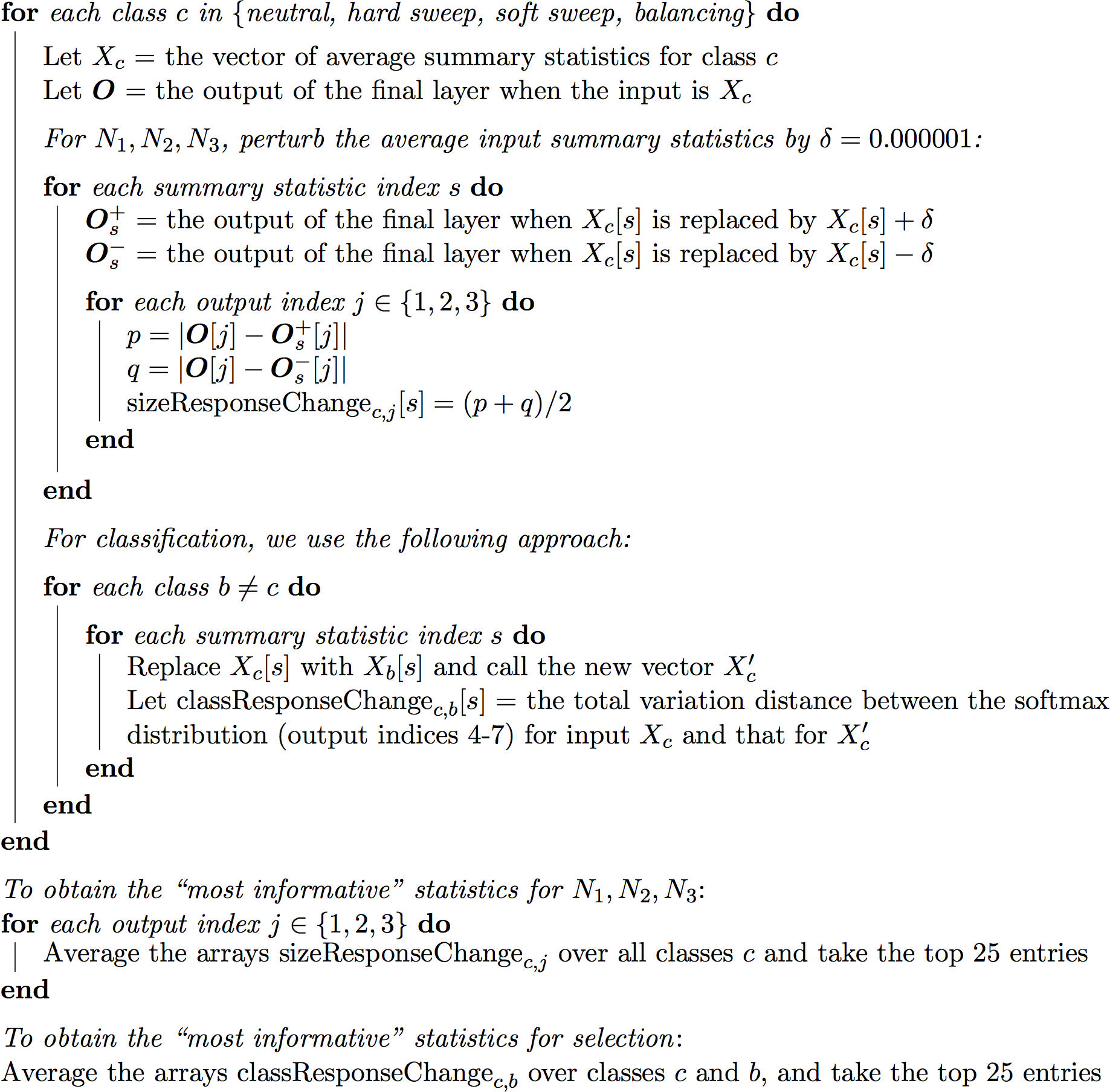

Supplement: S1 Algorithm — (TIFF) [file pcbi.1004845.s009.tiff]
